# CRISPR/Cas9 delivery by NIR-responsive biomimetic nanoparticles for targeted HBV therapy

**DOI:** 10.1186/s12951-021-01233-4

**Published:** 2022-01-06

**Authors:** Dan Wang, Ling Chen, Chengbi Li, Quanxin Long, Qing Yang, Ailong Huang, Hua Tang

**Affiliations:** 1 Key Laboratory of Molecular Biology for Infectious Diseases (Ministry of Education), Institute for Viral Hepatitis, Department of Infectious Diseases, The Second Affiliated Hospital, Chongqing Medical University, 1 Yi Xue Yuan Road, Chongqing, 400016 China; 2 The People’s Hospital of Rongchang District, Chongqing, 402460 China

**Keywords:** CRISPR/Cas9, UCNP, Biomimetic nanoparticles, On-demand release, HBV, Antiviral therapy

## Abstract

**Background:**

Currently, there are no curative drugs for hepatitis B virus (HBV). Complete elimination of HBV covalently closed circular DNA (cccDNA) is key to the complete cure of hepatitis B virus infection. The CRISPR/Cas9 system can directly destroy HBV cccDNA. However, a CRISPR/Cas9 delivery system with low immunogenicity and high efficiency has not yet been established. Moreover, effective implementation of precise remote spatiotemporal operations in CRISPR/Cas9 is a major limitation.

**Results:**

In this work, we designed NIR-responsive biomimetic nanoparticles (UCNPs-Cas9@CM), which could effectively deliver Cas9 RNP to achieve effective genome editing for HBV therapy. HBsAg, HBeAg, HBV pgRNA and HBV DNA along with cccDNA in HBV-infected cells were found to be inhibited. These findings were confirmed in HBV-Tg mice, which did not exhibit significant cytotoxicity and minimal off-target DNA damage.

**Conclusions:**

The UCNPs-based biomimetic nanoplatforms achieved the inhibition of HBV replication via CRISPR therapy and it is a potential system for efficient treatment of human HBV diseases.

**Graphical Abstract:**

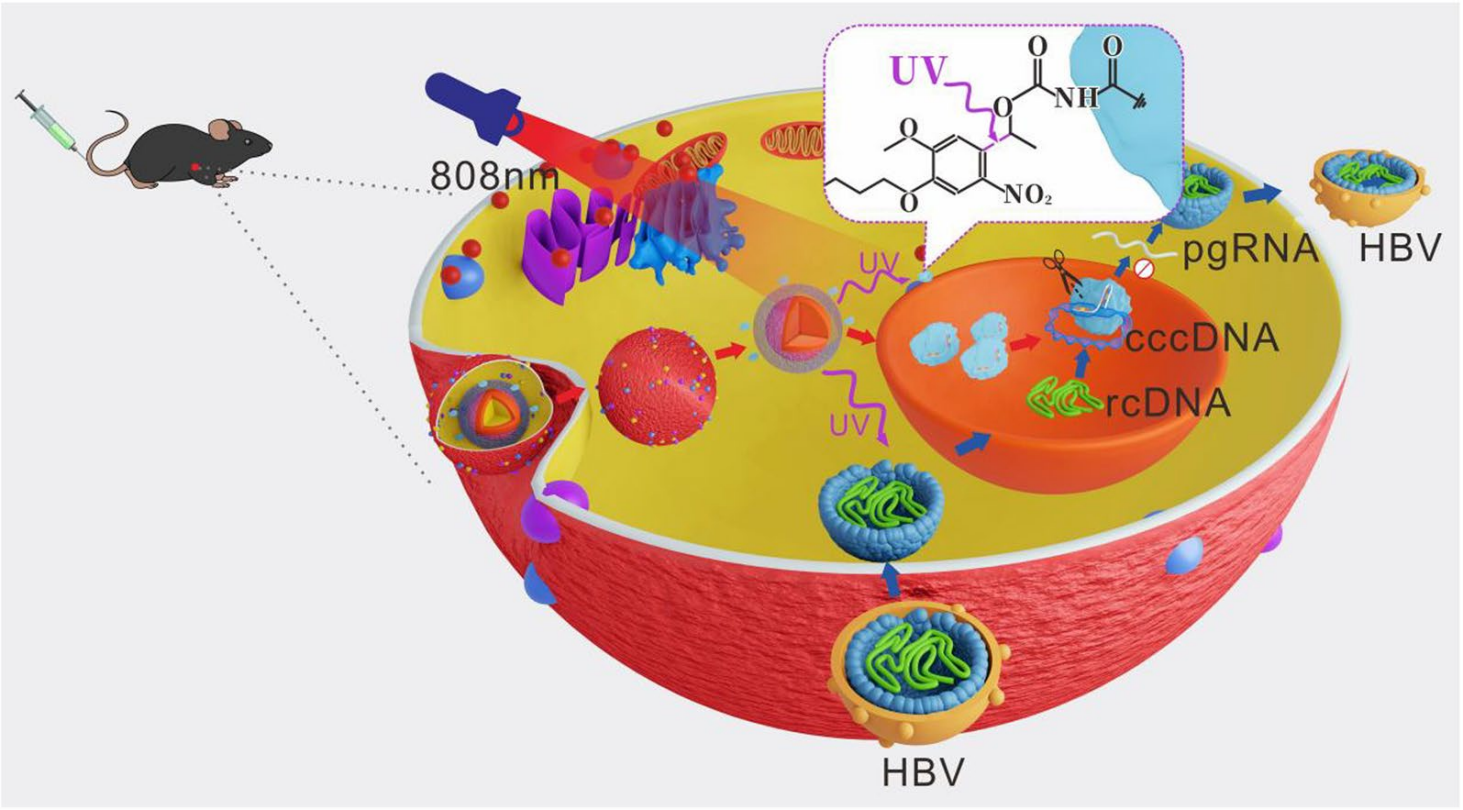

**Supplementary Information:**

The online version contains supplementary material available at 10.1186/s12951-021-01233-4.

## Background

Globally, more than 300 million persons worldwide are chronically infected with hepatitis B virus (HBV), which is associated with about 1 million annual deaths as a result of liver diseases [1]. Currently, the approved HBV therapies are mainly based on interferons (IFNs) and nucleoside analogs (NUCs). However, lengthy treatment is associated with drug resistance mutations and a risk of Hepatic Fibrosis/Cirrhosis. Moreover, these drugs cannot target or eliminate HBV covalently closed circular DNA (cccDNA), which inhibits complete neutralization of chronic hepatitis B (CHB) infections [2, 3]. Currently, studies on HBV cccDNA targeted therapy are focused on two aspects: i. Inhibiting the formation and persistence of cccDNA, for example, using targeted viral proteins HBx and HBc to negatively regulate cccDNA formation. ii. Elimination of cccDNA through epigenetic modifications, such as histone modifications and methylation of HBV cccDNA, or gene-editing therapies, such as Clustered Regularly Inter Spaced Palindromic Repeats (CRISPR) and CRISPR-associated protein 9 (CRISPR/Cas9) [4–6]_._ Among these strategies, CRISPR/Cas9 is currently the most promising therapeutic means [7–9].

Studies on the preferential matches of viral genes to CRISPR spacer sequences have shown that efficient and coordinated expressions of viral genes are critical for viral replication and lysis [10, 11]. In 2013, Prof. Zhang published a new method for gene knockout in vitro based on CRISPR/Cas9 technology [12]. Researchers then reported that CRISPR/Cas9 could specifically cut the HBV genome and inhibit HBV infection in the HepG2-NTCP cell models [13, 14]. In 2015, Wang et al. found that CRISPR/Cas9 focuses on common conserved regions of HBV polygenic subtypes to disrupt HBV cccDNA [15]. Then, using immunodeficient mice that mimick HBV chronic infections, Zhu et al. found that CRISPR/Cas9 significantly suppressed HBV replication levels [16]. Subsequently, Wang et al. constructed a gRNA-miR-HBV-gRNA triple expression framework, which significantly enhanced the clearance efficiency of HBV cccDNA [3]. Kostyusheva also found that some small molecule inhibitors of DNA repair increase the efficiency of CRISPR/Cas9 in eliminating HBV cccDNA [17]. In 2020, Yang et al. demonstrated that CRISPR/Cas9-mediated non-cutting editing strategy in the base-editing system could permanently inactivate the HBV genome in vitro, while avoiding off-target effects [18]. These studies show that the CRISPR/Cas9 can directly disrupt HBV cccDNA, providing a new strategy for hepatitis B cure. However, CRISPR/Cas9 therapy is limited by ineffective targeted delivery. Viral delivery strategies, such as Adeno-Associated Virus (AAV), have been used to deliver CRISPR/Cas9 vectors in vivo. However, viral vectors may lead to oncogenic effects, toxicity, immunogenicity and insertional mutagenesis. In addition, due to the low packaging capacity of AAV, viral titers required for therapeutic efficacy are usually several orders of magnitude higher than acceptable clinical limits [19, 20]. Non-viral vector delivery methods have the potential to overcome these limitations. Several vehicles, including cell-penetrating peptides (CPPs) [21], metal-organic cages [22]. polymeric and lipid-based nanoparticles [23–26], DNA nanoclews [27], graphene oxide [28], “core–shell” artificial viruses and other nanomaterials [29, 30] have been shown to be excellent for Cas9/sgRNA delivery. However, temporal and spatial remote control of on-demand CRISPR/Cas9 release is still elusive.

Photoregulation has been proved to be a promising non-invasive strategy [31, 32]. Upon irradiation with UV or visible light, several photocaged molecules, such as spiropyran, diazobenzene, 2-nitrobenzyl and its derivatives, are prone to structural exchanges or ester bond cleavage [33]. However, their biological applications are limited by the low energy and penetration force of UV-visible light. Among these light stimuli, near-infrared (NIR) light (650–1350 nm) has a deeper penetration depth and is associated with less damage compared to UV-visible light. As NIR light nanotransducers, lanthanide upconversion nanoparticles (UCNPs) can convert NIR photons into UV or visible photons via anti-Stokes emission. UCNPs are easy to functionalize, are biocompatible, biodegradable, non-toxic, hypoimmunogenic and have excellent drug loading capacities [34–37]. Even though UCNPs have a therapeutic potential, bare UCNPs do not possess targeting properties. An improved tumor tissue-targeting ability and extended the blood-circulation time can be achieved by using surface functionalized nanoparticles [38–40]. Recently, strategies for drug delivery by bioinspired cell membrane coated nanoparticles have been developed. The top-down biomimetic approach is a potential route for endowing nanoparticles with colloidal stability, immune escape and homotypic targeting abilities to overcome the physiological barriers in vivo [41–43]. Cell-membrane-derived vesicles (CMs) can be derived from various cell membranes, including erythrocytes [44], leukocytes [45], platelets [46], tumor cells [47], mesenchymal-stem-cells [48] among others.

**Scheme 1:**
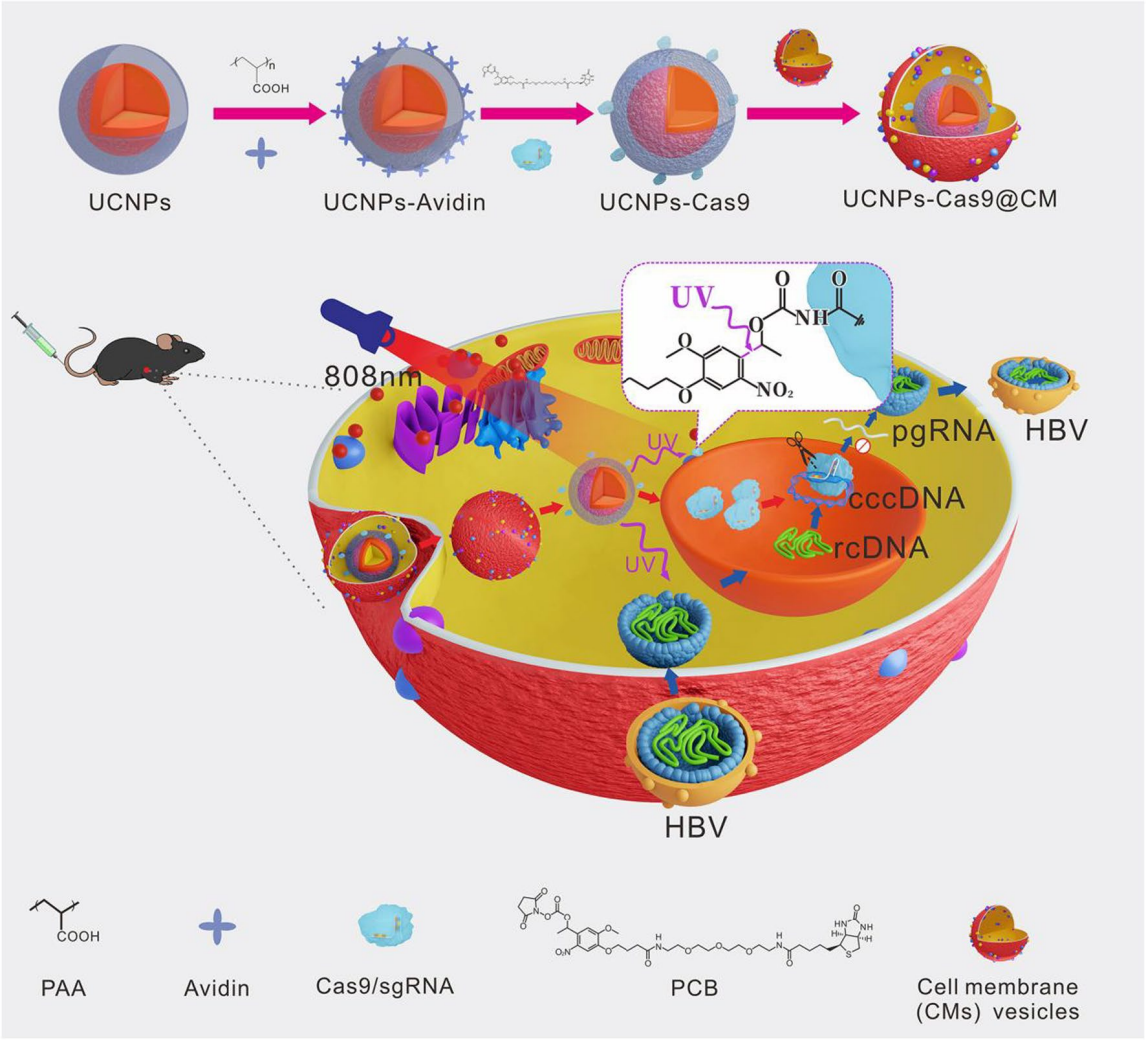
Schematic illustration of the NIR light-controlled biomimetic nanoplatform for the delivery of Cas9 RNP for HBV gene editing

In this study, we devised a biomimetic strategy for NIR-controlled CRISPR/Cas9 delivery system to realize effective genome editing in HBV-infected cells and HBV-Tg mice. To realize NIR laser-controlled gene editing, we assembled the UCNPs-Cas9 complex using the photolysable PC Biotin-NHS Ester (PCB). As shown in Scheme 1, the NIR-responsive biomimetic nanoparticles (UCNPs-Cas9@CM) were prepared in two sections: i. Acquistion of CMs from cognate host cells and ii. Coating CMs onto UCNPs-Cas9 surfaces. Accurate UCNPs-Cas9 delivery was achieved by the target homing effects of CMs. Then, Cas9/sgRNA was released under NIR light irradiation, entered the nucleus with the aid of nuclear localization sequence (NLS) to disrupt HBV cccDNA thereby inhibiting viral replication. By preparing a specific HBV-targeting sgRNA, we realized the inhibition of HBV progression through the biomimetic nanoplatform.

## Results and discussion

### Design and synthesis of NIR-controlled UCNPs-Cas9@CM

**Fig. 1:**
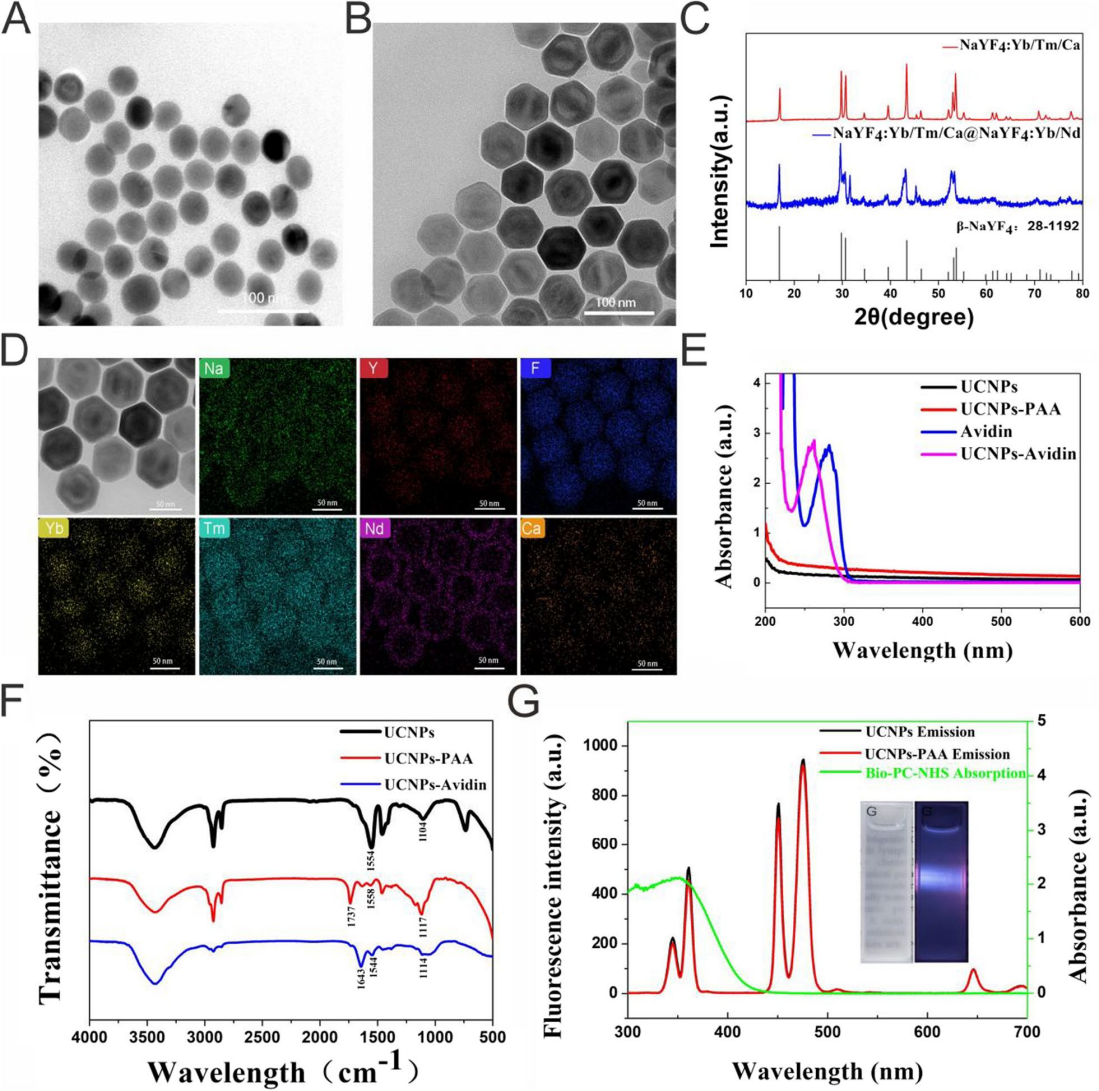
Characterization of UCNPs-Cas9. TEM images of **A** NaYF_4_:Yb/Tm/Ca, **B** NaYF_4_:Yb/Tm/Ca@NaYF_4_:Yb/Nd. **C** XRD patterns of NaYF_4_:Yb/Tm/Ca@NaYF_4_:Yb/Nd. a.u., arbitrary units. **D** NaYF_4_:Yb/Tm/Ca@NaYF_4_:Yb/Nd and its EDX elemental mapping. **E** UV–vis absorption spectrum of UCNPs, UCNPs-PAA, Avidin and UCNPs-Avidin. **F** FTIR spectra of UCNPs, UCNPs-PAA and UCNPs-Avidin. **G** Fluorescence spectrum of UCNPs and UCNPs-PAA activated by 808 nm laser and UV–vis absorption spectrum of PCB. Inset: bright-field and NIR fluorescent images of UCNPs

**Fig. 2:**
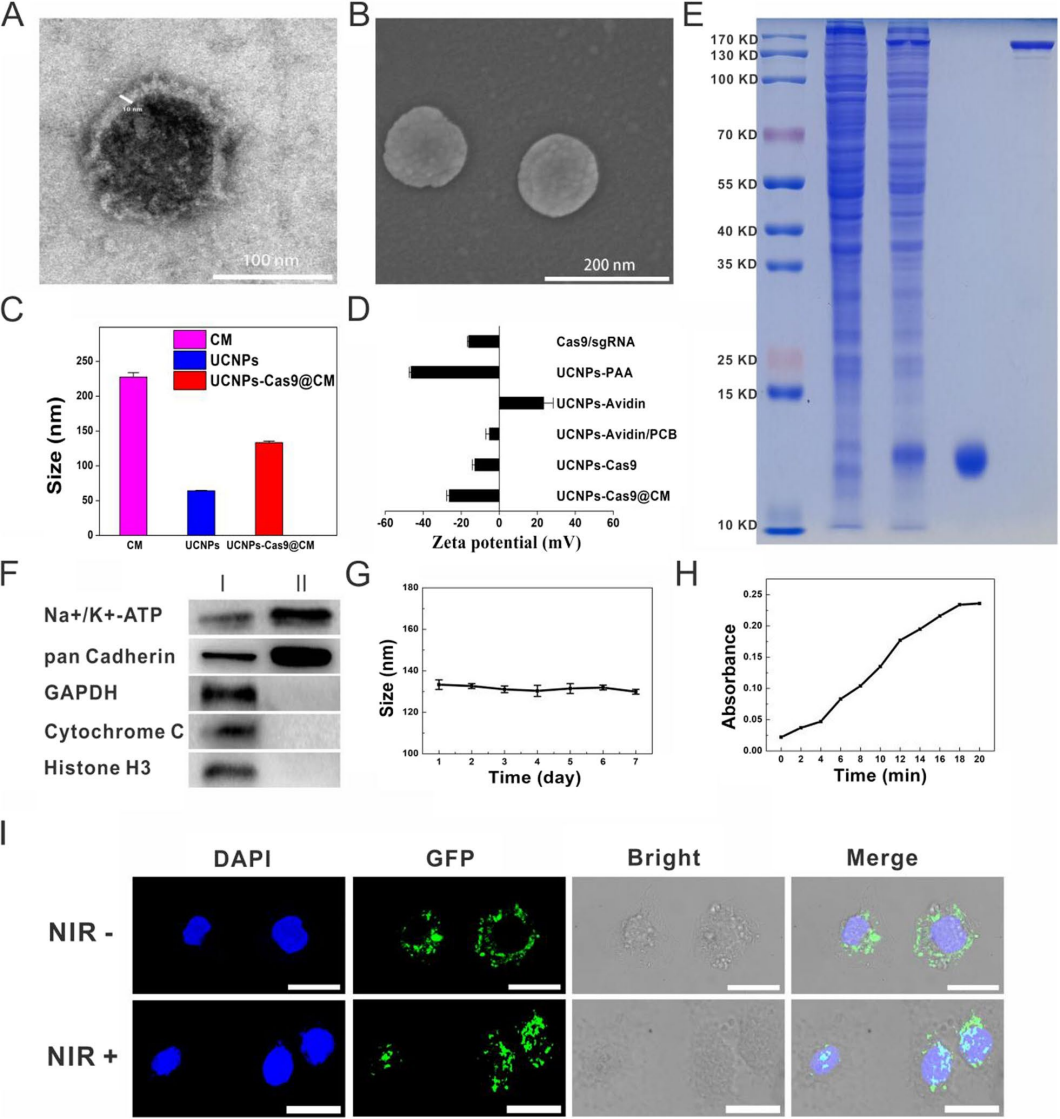
Characterization of NIR-controlled UCNPs-Cas9@CM. TEM (**A**) and SEM (**B**) images of UCNPs-Cas9@CM. **C** Hydrodynamic size distribution of CMs, UCNPs and UCNPs-Cas9@CM. **D** Zeta potentials of Cas9/sgRNA, UCNPs-PAA, UCNPs-Avidin, UCNPs-Avidin/PCB, UCNPs-Cas9 and UCNPs-Cas9@CM. **E** SDS-PAGE protein analysis of total cell proteins, UCNPs-Cas9@CM, avidin and Cas9 protein (36 pmol) (from left to right). Samples were stained with Coomassie Blue. **F** WB analysis of (I) cell lysate and (II) CMs. **G** Hydrodynamic diameter variations over time of UCNPs-Cas9@CM. **H** UV–vis absorption (280 nm) of the supernatant of UCNPs-Cas9 was measured using a 808 nm laser at different irradiation times. **I** CLSM images of cells were incubated with UCNPs-Cas9@CM at 6 h with NIR-/ + irradiation (green: GFP-labeled Cas9; blue: DAPI). Scale bars: 25 μm

We used an aliovalent Ca^2+^ ion doping strategy to obtain enhanced photoemission intensity under NIR light excitation, as previously reported [49]. First, we synthesized Ca-doped core NaYF_4_:Yb^3+^/Tm^3+^ particles with a mean diameter of 41 nm (Fig. 1A, Additional file 1: Fig. S1A-B). To achieve the responsiveness of 808 nm NIR excitation, a NaYF_4_:Yb/Nd shell was constructed on the core to form a core–shell structure of UCNPs with an average size of 64 nm (Fig. 1B, Additional file 1: Fig. S1C-D). High Resolution TEM (HRTEM) and selected area electron diffraction (SAED) revealed that lattice stripe spacing of UCNPs was 0.52 nm, corresponding to the typical (100) plane of the hexagonal structure (Additional file 1: Fig. S1E-F). Additionally, XRD also showed that the UCNPs have a hexagonal phase because their diffraction peaks were well indexed to the hexagonal β-NaYF_4_ (JCPDS Card No. 28–1192) (Fig. 1C). TEM mapping confirmed the composite structure of UCNPs (Fig. 1D). These results implied that NaYF_4_:Yb/Tm/Ca@NaYF_4_:Yb/Nd Core–shell UCNPs had been successfully synthesized. Water-soluble carboxyl-functionalized nanoparticles (UCNPs-PAA) were obtained after treatment with PAA. The UCNPs-PAA were conjugated onto avidin proteins through amide bonds to synthesize UCNPs-Avidin. These surface modifications were confirmed by UV–Vis spectra, FTIR spectra (Fig. 1E, F) and ζ potential measurements (Fig. 2D). The main UV-absorption maximum in avidin blue-shifted after being decorated with UCNPs-PAA. Visible absorption peaks for FTIR at 1737 cm^−1^ and 1558 cm^−1^ indicated stretching vibrations of C = O and COO^−^ groups, implying effective modifications of the carboxyl group. The N–H bending vibrations were also observed at 1643 cm^−1^ (amide). Moreover, ζ potential of UCNPs-PAA was -46.46 ± 0.89 mV, however, after avidin modification, it increased to 22.02 ± 4.95 mV. These results imply that UCNPs-Avidin had been successfully synthesized. Compared to UCNPs, fluorescence intensities of UCNPs-PAA did not decrease. Moreover, UV-vis absorption of PCB and emission spectrum of UCNPs partially overlapped (Fig. 1G). PCB, a photocleavable linker with UV-irradiation, was used to couple UCNPs-Avidin and Cas9 RNP to address NIR-responsive CRISPR/Cas9 for controllable gene editing. Due to the strong affinity between avidin and biotin, PCB could easily bind UCNPs-Avidin to form UCNPs-Avidin/ PCB nanoparticles, which were covalently conjugated with Cas9 proteins and incubated with sgRNA to generate UCNPs-Cas9. Changes in ζ potentials confirmed the success of each step.

Three HBV-specific gRNAs (RT, 6 and 17) were selected from published literature [14, 16, 53] and synthesized in vitro using T7 polymerase. The in vitro cleavage assay showed that sgRNA17 was better at targeting the HBV gene than other sgRNAs (Additional file 1: Fig. S2). Thus, sgRNA17 was selected for subsequent experiments.

HepG2.2.15 and HepAD38 cell lines, which had been developed at Chongqing Medical University, were selected as HBV replication models, and cell membrane fragments obtained for further experiments (See Additional file 1: Fig. S3 for CMs characterization). To prepare UCNPs-Cas9@CM, UCNPs-Cas9 were cloaked with CMs by extrusion through polycarbonate membranes with reducing pore sizes (~ 400 nm, ~ 200 nm). The obtained UCNPs-Cas9@CM were resuspended in PBS. TEM imaging revealed a spherical shape for the core–shell structure and an outer shell that was about 10 nm in thickness. Surface morphologies were examined by SEM (Fig. 2A–B). Moreover, particle sizes and ζ potentials were determined (Fig. 2C–D). Protein ingredients of UCNPs-Cas9@CM was evaluated by coomassie brilliant blue staining. Membrane proteins within the source cell membrane were widely retained, and there were bands corresponding to monomeric avidin (17 kDa) and Cas9 (160 kDa), implying that avidin and Cas9 had been successfully embedded in biomimetic nanoparticles (Fig. 2E). Notably, Cas9 protein band in 10 μl particle solution was about 1/3 of pure Cas9 protein (36 pmol) band. That is, every 10 μl of UCNPs-Cas9@CM solution contained about 12 pmol Cas9. Western blot showed that pan cadherin and Na^+^/K^+^-ATPase (membrane markers) were well retained, while compared to GAPDH (cytosolic marker), Cytochrome C (mitochondrial marker) and Histone H3 (nuclear marker), implying selective retention of membrane proteins (Fig. 2F). Consequently, these findings indicated that the UCNPs-Cas9 nanoparticles had successfully adhered to CMs. The UCNPs-Cas9@CM exhibited good stabilities, and hydrodynamic diameter variation was almost constant over 7 days (Fig. 2G).

**Fig. 3:**
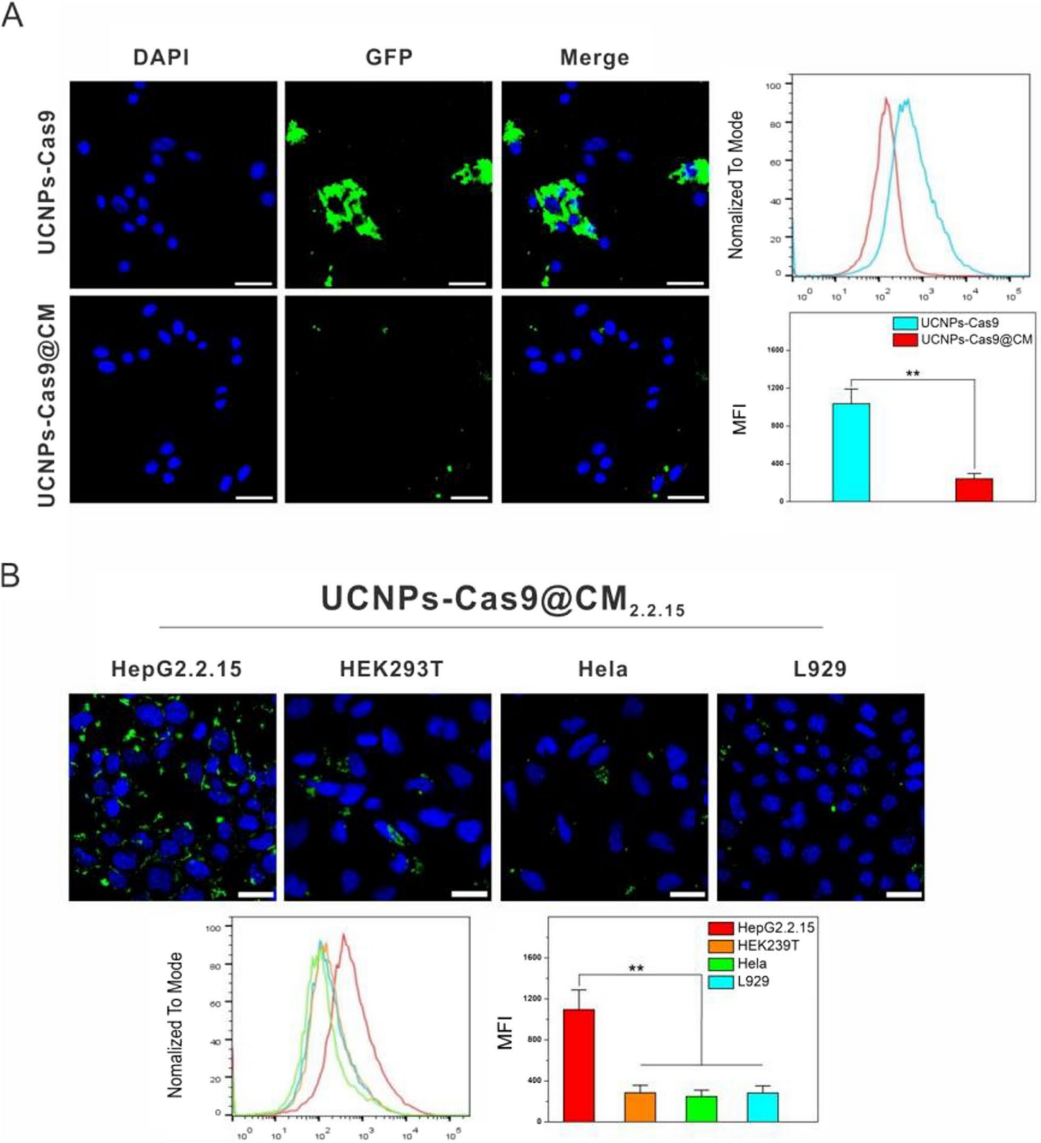
Homotypic target and immune escape capacity of UCNPs-Cas9@CM. **A** CLSM images and flow cytometry profiles of RAW264.7 cells upon 6 h of co-culture with UCNPs-Cas9 or UCNPs-Cas9@CM. **B** CLSM images and flow cytometry profiles of HepG2.2.15, HEK293T, Hela, L929 cells after 6 h of co-culture with UCNPs-Cas9@CM_2.2.15_. Scale bars: 25 μm

Effectiveness of NIR release was also examined. The UCNPs-Cas9 solution was either exposed or not, to NIR irradiation. Then absorption spectra of the supernatants were measured after centrifugation. As shown in Fig. 2H, the UV–vis absorbance at 280 nm was increased with the increasing NIR irradiation time, while the trend was not observed in NIR(-) group. Moreover, we had added another 2 control groups as NIR-responsive negative groups: (i) Replace UCNPs with Si-nanoparticles (noted as Si-Cas9, control particles group); (ii) Replace PCB with 3,3'-Thiodipropionic acid (TDPA) (noted as UCNPs-TDPA-Cas9, control linker group). While the Si-Cas9 and UCNPs-TDPA-Cas9 were exposed to NIR laser, no observable absorption changed at the wavelength of 280 nm was detected. The supernatant after centrifugation along with sgRNA couldn’t cleave the fragment that contain sgRNA target sequences (Additional file 1: Fig. S4). The results showed that neither Si-Cas9 nor UCNPs-TDPA-Cas9 could release Cas9 protein in respond to NIR irradiation. Next, we verified the above results in HepG2.2.15 cells. After co-culturing with the UCNPs-Cas9@CM for 3 h, the GFP-labeled Cas9 was localized in the cytoplasm. In addition, Figure S5A showed the overlapping co-location of Dil-labeled red CM and LysoTracker Green after 3 h co-culture of cells with the nanosystem, partly suggesting UCNPs-Cas9@CM were more likely taken up via endocytic pathway. Upon NIR irradiation, the fluorescence of GFP in UCNPs-Cas9@CM was observed in the nucleus at 6 h, while it only appeared in the cytoplasm in the control group (without NIR) (Fig. 3I, Additional file 1: Fig. S5B–C). Z-stack images also showed the same results (Additional file 1: Fig. S6). Our findings indicated that the nanocarriers had the potential for temporal and spatial remote control of on-demand CRISPR/Cas9 release.

### Immune escape and homotypic target of UCNPs-Cas9@CM in vitro

Ideally, a successful nanodelivery system should exhibit low immunogenicity and good targeting effects. Macrophages recognize proteins on cell surfaces to determine whether they need to be phagocytosed [54–57]. Antiphagocytic capacities of UCNPs-Cas9@CM against mouse macrophages (RAW 264.7) were investigated, using UCNPs-Cas9 as the control. After 6 h of co-culture, cell fluorescence showed that RAW264.7 cells internalized a large amount of UCNPs-Cas9. In comparison, RAW264.7 cells co-cultured with UCNPs-Cas9@CM showed very weak fluorescence signals. In addition, internalization efficiencies were evaluated by flow cytometry, and findings were in accordance with the above results (Fig. 3A, Additional file 1: Fig. S7). After respective incubations with PBS, LPS and UCNPs-Cas9@CM for 24 h, mRNA expression levels of inflammatory factors, such as tumor necrosis factor alpha (TNF-α), IL-1β and IL-8, were evaluated in RAW264.7 cells. Compared to the group treated with PBS and LPS, cytotoxicity was negligible in the UCNPs-Cas9@CM treated groups (Additional file 1: Fig. S8). These results implied that CMs-coated particles could effectively inhibit macrophage uptake.

To verify the homotypic targeting abilities of UCNPs-Cas9@CM to hepatic cells, cellular internalization of HepG2.2.15 cell membrane coated UCNPs-Cas9 NPs (termed UCNPs-Cas9@CM_2.2.15_) was evaluated in HepG2.2.15, HEK293T, HeLa, and L929 cells. UCNPs-Cas9@CM_2.2.15_ were shown to preferentially accumulate in HepG2.2.15 cells and exhibited a higher green fluorescence intensity. Conversely, the heterotypic cell groups exhibited weaker fluorescence intensities. Then mean fluorescence intensity (MFI) for each cell was measured by flow cytometry. Data was approximated to 3.9 ~ 4.5 folds in terms of MFI inside HepG2.2.15 cells compared to others (Fig. 3B). As time went by, cell internalization was faster (Additional file 1: Fig. S9). These findings suggested a high targeting specificity of UCNPs-Cas9@CM to cells.

### Therapeutic effects of UCNPs-Cas9@CM in vitro

Given that UCNPs-Cas9@CM could not damage normal liver cells and the NIR-responsive photothermal property, we assessed temperature changes by irradiating the UCNPs-Cas9@CM solution (Additional file 1: Fig. S10). Therefore, we used short interval irradiation (2 min break after 1 min irradiation) over the course of the experiment. Before using UCNPs-Cas9@CM to perform NIR-activated editing, a set of cell viability tests were performed. Viabilities of HepG2.2.15 and HepAD38 cells were not affected by different NIR powers for 20 min and illumination times for 2 W/cm^2^, respectively. Subsequently, effects of UCNPs-Cas9@CM at various concentrations on cell viability were evaluated. Finally, cells were incubated with various concentrations of UCNPs-Cas9@CM at a fixed laser power and illumination time (Additional file 1: Fig. S11). There were no significant effects on cell viability.

**Fig. 4:**
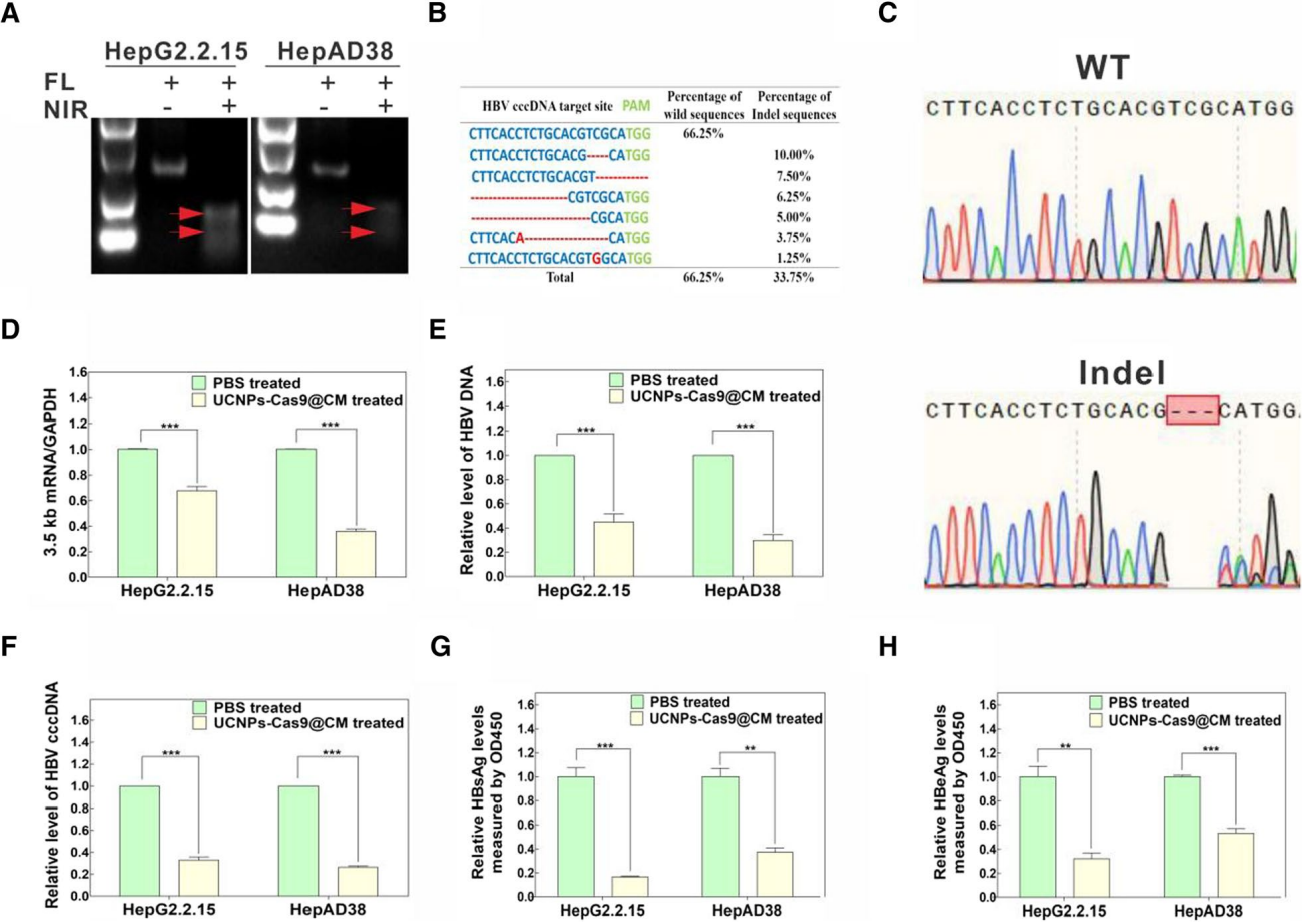
In vitro effects of UCNPs-Cas9@CM on HBV gene editing. **A** T7E1 assay for editing HBV in HepG2.2.15 and HepAD38 cells after UCNPs-Cas9@CM treatment with/without NIR Irradiation. **B** Sequence analysis of PCR amplicons of HBV cccDNA from HepG2.2.15 cells after UCNPs-Cas9@CM treatment with NIR irradiation. **C** Representative images of sanger sequencing profiles (from top to bottom) represent the wild type and indel type, respectively. **D–F** qRT-PCR analysis of HBV 3.5 kb mRNA, intracellular HBV DNA and HBV cccDNA levels in HepG2.2.15 and HepAD38 cells after UCNPs-Cas9@CM treatment with NIR irradiation, PBS treatment was used as the control. **G–H** HBsAg and HBeAg levels in supernatants of HepG2.2.15 and HepAD38 cells after UCNPs-Cas9@CM treatment with NIR irradiation, PBS treatment was performed as a control. Data are shown as mean ± SD, **p < 0.01, ***p < 0.001

We also determined whether UCNPs-Cas9@CM could effectively disrupt HBV cccDNA in cell models. HepG2.2.15 and HepAD38 cells were cultured with UCNPs-Cas9@CM for a further 4 h before NIR irradiation. After 3 days, genomic DNA was isolated from cells and the T7E1 assay was performed. Figure 4A showed an excellent genetic editing efficiency in vitro. Moreover, PCR amplicons from UCNPs-Cas9@CM + NIR treatment groups exhibited DNA sequencing double peaks, confirming indels of the HBV cccDNA locus (Fig. 4B–C, Additional file 1: Fig. S12). Next, we assessed the first few putative off-target sites for target sequences in Homo sapiens. The off-target effect of UCNPs-Cas9@CM in vitro was negligible (Additional file 1: Fig. S13). Overall, sgRNA17 could specifically recognize and edit the HBV cccDNA. Thereafter, we evaluated the potential of UCNPs-Cas9@CM for NIR-activated editing of HBV cccDNA in HepG2.2.15 and HepAD38 cells. Cells were subjected to UCNPs-Cas9@CM + NIR treatment. We then measured HBV 3.5 kb RNA, intracellular HBV DNA and HBV cccDNA levels by qRT-PCR (Fig. 4D–F). At the same time, HBV viral antigens (like HBsAg and HBeAg) in supernatants were determined by ELISA (Fig. 4G–H). HBV 3.5 kb RNA, intracellular HBV DNA, HBV cccDNA and HBV viral antigens were significantly decreased after incubation with UCNPs-Cas9@CM, relative to the control group.

### Antiviral effects of UCNPs-Cas9@CM in vivo

**Fig. 5:**
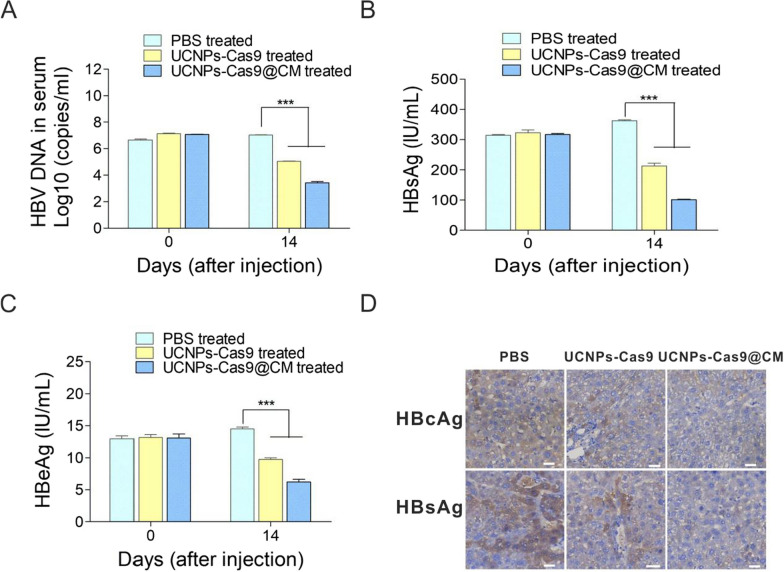
In vivo effects of the UCNPs-Cas9@CM on HBV gene editing. **A** Serum HBV DNA load in HBV-Tg mice sera were measured by qRT-PCR. Levels of HBsAg (**B**) and HBeAg (**C**) in mice serum were measured by CLIA. **D** Representative images of intrahepatic expression levels of HBsAg and HBcAg in HBV-Tg mice after 14 days of different treatments (IHC staining, scale bars: 30 μm). For **A**–**C**, data are presented as mean ± SD, n = 3 in each group. ***p < 0.001

The NIR-controlled UCNPs-Cas9@CM system could uniquely suppress HBV replication in vitro. We then evaluated the ability of the UCNPs-Cas9@CM system to repress HBV replication in vivo using the HBV-Tg mice model. In in vivo study, UCNPs-Cas9 were coated with mouse liver cell membrane fragments to form the UCNPs-Cas9@CM nanoparticles. To validate the homing capability of UCNPs-Cas9@CM in vivo, we administered intravenous (100 µl, 5 mg/ml) and detected the biodistribution of UCNPs-Cas9@CM by ICP-MS after 24 h. The liver had the highest %ID/g of UCNPs (Additional file 1: Fig. S14A). In the later, UCNPs-Cas9@CM was labeled with DiR dye and the distribution of particles was determined over time by NIR imaging. As shown in Additional file 1: Fig. S14B, the UCNPs-Cas9@CM were observed to accumulate in the liver from 0.5 h to 24 h after injection. Both ICP-MS analysis and bioluminescence imaging revealed that CMs-functionalized UCNPs-Cas9@CM mainly distributed in the liver as expected. These results suggested that UCNPs-Cas9@CM had a good homing capability in vivo. Next, we performed a hemolysis assay to assess the safety of UCNPs-Cas9@CM. There was no hemolysis after treatment with different UCNPs-Cas9@CM concentrations (Additional file 1: Fig. S15A). Nine HBV-Tg mice with elevated HBV DNA, HBsAg and HBeAg levels were selected and randomized into three groups of three mice each. Then, mice were injected with PBS, UCNPs-Cas9 or UCNPs-Cas9@CM for three consecutive days after which their livers were irradiated with a NIR laser for 30 min. During treatment, their body weights were monitored every 2 days after injection. Mice were sacrificed on day 14 post-injection by eye bloodletting, cervical dislocation, after which their livers were collected. Compared with the UCNPs-Cas9 group, the UCNPs-Cas9@CM group significantly decreased serum levels of HBV DNA, HBsAg and HBeAg, as well as the HBsAg and HBcAg levels in hepatocytes (Fig. 5A–D). However, there were no significant changes in various blood parameters and body weights (Additional file 1: Fig. S15B–C). To further analyze the excretion of UCNPs-Cas9@CM in vivo, excreted concentrations of lanthanide ion (Y^3+^) from fecal and renal excretions were measured using ICP-MS. As shown in Additional file 1: Fig. S15D, Y^3+^ levels steadily decreased over time in mice urine and feces, implying that UCNPs were efficiently excreted. Major organs from mice in different groups were also obtained and fixed for histology analysis to assess the toxicity effects of UCNPs-Cas9@CM-based NIR laser-controlled gene editing. H&E staining of the organs showed that there were no significant differences after the treatments (Additional file 1: Fig. S15E). These results confirmed that UCNPs-Cas9@CM had good biocompatibility and properties without systemic toxic effects. Finally, genomic DNA extracted from mice livers was used to evaluate the off-target effects and PCR amplicons containing the off-target sites were validated by T7E1 assays. No off-target effect was detected (Additional file 1: Fig. S16). Overall, our nanoparticles achieved anti-HBV therapy via NIR-controlled CRISPR/Cas9 system release.

## Conclusions

By exploiting the principle of immune escape and homotypic targeting ability of CMs, we achieved accurately UCNPs-Cas9 delivery to hepatocytes. When applying NIR stimulation, UCNPs emitted UV light and triggered the cleavage of photocaged linkers. Consequently, Cas9/sgRNA could be released to enter the nucleus with the help of NLS. Guided by HBV-specific sgRNA, our platform successfully inhibited HBV replication in vitro and in vivo. Moreover, since our gRNA targeted the viral genome rather than the human genome, it was the best way to functionally inactivate HBV cccDNA. The significance of CRISPR/Cas9 in eliminating cccDNA from chronic HBV infection should be clinically confirmed. Our treatment platform offers a strategy for the development of CRISPR therapeutics, especially those targeting infectious diseases.

## Materials and methods

### Materials and reagents

LnCl_3_·6H_2_O (Ln = Y, Yb, Tm and Nd, 99%), Oleic acid (OA, 85%), 1-Octadecene (ODE, 90%), Cyclohexane (99%) and Poly (acrylic acid) (MW 2000) (PAA) were purchased from Aladdin, Inc (Shanghai, China). N-hydroxysuccinimide (NHS), N-(3dimethylaminopropyl)-N’-ethylcarbodiimide hydrochloride (EDC) and avidin were obtained from Sangon Biotech Co., Ltd (Shanghai, China). Polycarbonate porous membrane and Avanti mini extruder were obtained from Avanti Polar Lipids. The RIPA lysis buffer, cell counting kit-8 (CCK-8), phenylmethanesulfonyl fluoride (PMSF) and the lipopolysaccharide (LPS, O55:B5) of *E.coli* were purchased from Beyotime Biotechnology (Beijing, China). PCB (Sigma-Aldrich, USA), T7 endonuclease 1 (T7E1), NLS-Cas9-EGFP and NLS-Cas9-NLS (Genscript Biotech, China) were commercially obtained. HPLC-purified oligonucleotides (Additional file 1: Table S1) were bought from Tsingke Biotechnology Co., Ltd. (Beijing, China). All chemicals were analytical grade. The medium (DMEM), fetal bovine serum (FBS) and antibiotics were purchased from Gibco (Rockville, MD). Aqueous solutions were prepared using Milli-Q water (18.2 MΩ, Millipore, USA).

### Transcription and purification of sgRNA

T7 RNA polymerase was used to transcribe sgRNAs in vitro from the DNA template. Briefly, sgRNA was synthesized using HiScribe T7 Quick High Yield RNA Synthesis Kit (NEB, #E2040S), followed by incubation with DNase I (Takara, #2270A) for 15 min at 37 °C to remove template DNA. The resulting RNA were purified using a HiPure RNA Pure Micro Kit (Magen, #R2144). The obtained sgRNA was measured using a Nano-500 spectrophotometer (Allsheng, China) and preserved at − 80 °C until further use.

### Efficient screening of sgRNA in vitro

SgRNA target sequences were amplified by PCR using the PCH9/3091 plasmid as a template. Briefly, 15 μl of the reaction system, including 50 ng Cas9, 100 ng sgRNA and 2 μl 10 × Reaction Buffer, was preincubated at 37 °C for 10 min. Then, 5 μl of the PCR fragment was added followed by incubation at 37 °C for 2 h. Then, 10 μl/sample were analyzed by 1.5% agarose gel electrophoresis.

### Preparation of Ca-oleate precursor

NaOA (6.09 g) was dissolved in 20 ml ethanol and mixed with 10 mL Ca(NO_3_)_2_ solution (10 mM) in a round-bottom flask. Then, 35 ml cyclohexane was added followed by heating at 80 °C for 4 h under reflux. After completion, the mixture was cooled to room temperature (RT) and transferred into a separating funnel. The upper layer was obtained and washed several times. Finally, residual cyclohexane was removed by rotary evaporation to obtain a white Ca-oleate solid complex.

### Synthesis of Ca^2+^ doped core NaYF_4_:Yb^3+^/Tm^3+^

NaYF_4_:Yb/Tm/Ca nanoparticles were prepared as previously reported [49]. 0.5 mM core precursor LnCl_3_ aqueous solution (including 0.1205 g YCl_3_•6H_2_O, 0.03874 g YbCl_3_•6H_2_O, 0.000958 g TmCl_3_•6H_2_O) were mixed and heated to 100 °C for 30 min. Next, Ca-oleate (30 mg), OA (3.75 mL) and ODE (7.5 mL) were added and heated to 150 °C for 1.5 h under constant stirring, and then cooled to RT. Then, a methanol solution (5 mL) containing NaOH (0.05 g) and NH_4_F (0.074 g) was added and stirred at 50 °C for 30 min. The solution was subsequently heated to 100 °C for 30 min, followed by heating to 300 °C for 1.5 h. After the solution had been cooled to RT, absolute ethanol was added to precipitate the product. The product was washed using ethanol/cyclohexane (1:1 v/v), and finally dispersed in 4 mL cyclohexane for further use. All experiments were performed under argon.

### Synthesis of NaYF_4_:Yb/Tm/Ca@NaYF_4_:Yb/Nd core–shell UCNPs

A 0.4 mM core–shell precursor LnCl_3_ aqueous solution (including 0.04852 g YCl_3_•6H_2_O, 0.07172 g NdCl_3_•6H_2_O, 0.01549 g YbCl_3_•6H_2_O) was mixed and heated to 100 °C for 30 min. Then, OA (3.75 mL) and ODE (7.5 mL) were added and heated to 150 °C for 1.5 h followed by cooling to RT. Thereafter, 4 mL of the pre-prepared core solution and a methanol solution (5 mL) containing NaOH (0.04 g) and NH_4_F (0.0592 g) were added and stirred at 50 °C for 30 min. The solution was subsequently heated to 100 °C for 30 min, followed by heating to 300 °C for 1.5 h. After the solution had been cooled to RT, absolute ethanol was added to precipitate the product. The product was washed using ethanol/cyclohexane (1:1 v/v), and finally dried at 60 °C overnight. All experiments were performed under argon.

### Preparation of UCNPs-Cas9

Oleic-UCNPs were converted to hydrophilic UCNPs before coupling with avidin. Briefly, PAA (0.5 g) and diethylene glycol (10 mL) were heated to 110 °C for 1 h. Then, 2 mL of cyclohexane containing 30 mg oleic-UCNPs was added and maintained at 110 °C for another 1 h after which the mixture was heated to 240 °C for 1 h and left to cool to RT. Excess diluted hydrochloric acid (pH 4–5) was added, followed by vortexing and centrifugation. Resulting nanoparticles were marked carboxyl-group-functionalized UCNPs (UCNPs-PAA). As previously described [50, 51], EDC/NHS covalent chemistry was used to bind avidin to UCNPs-PAA surface. Briefly, 1 mg UCNPs-PAA, 50 mg EDC and 25 mg NHS were added in 1 mL of PBS (pH 5.5) and incubated at 37 °C for 1 h. Then, 1 mg avidin was immediately added and the pH adjusted to 8. The reaction solution was incubated at 4 °C overnight. The obtained nanoparticles were washed, resuspended in 600 μl PBS and dissolved in the prepared PCB solution in 400 μl PBS. The reaction solution was rotated for 2 h at 37 °C. Then, particles were collected and washed. Meanwhile, equimolar ratios of NLS-Cas9-EGFP and sgRNA were mixed in PBS for 10 min at RT to form Cas9 RNP. The Cas9 RNP and the obtained particles were mixed and incubated overnight at 4 °C under rotation. Dialysis was performed on the mixture using an ultrafiltration spin column (Vivaspin 500, 300KD, #RT-VS0152-5) to discard the unbound Cas9 RNP. The obtained UCNPs-Cas9 was resuspended in 500 μl of PBS and stored at 4 °C for futher use.

### Preparation of cell membrane fragments

HepG2.2.15 and HepAD38 cell membranes were obtained using a Membrane Protein Extraction Kit (Beyotime Biotechnology, #P0033). Briefly, cells were harvested and resuspended in an ice-cold membrane protein extraction buffer solution for 15 min. Then, cells were lysed by freeze-thawing and the homogenate centrifuged (700 g × 15 min, 4 °C). The remaining supernatant was collected and further centrifuged (13,300 g, 30 min, 4 °C) to obtain CMs which were freeze-dried, weighed and stored at -80 °C.

### Preparation and characterization of UCNPs-Cas9@CM

Firstly, lyophilized CMs were resuspended in PBS buffer prior to use. Next, equal masses of UCNPs-Cas9 and CMs were mixed and sequentially extruded through a polycarbonate membrane of 400- and 200- nm pore sizes for a total of 21 times using an Avanti mini extruder, respectively. Finally, UCNPs-Cas9@CM were obtained by centrifugation and suspended in PBS.

Fluorescence and UV–vis absorption spectra were determined using a Cary Eclipse Fluorescence Spectrophotometer (Agilent Technologies, CA) and a UV-2550 UV–vis spectrophotometer (Shimadzu, Japan), respectively. Nanoparticles were characterized by transmission electron microscopy (TEM, Talos F200X, FEI), scanning electron microscopy (SEM, SU8000, Hitachi), a laser particle size and zeta system (NanoBrook Omni, Brookhaven), Thermo Fisher Scientific Nicolet IS10 Fourier Transform infrared spectroscopy (FTIR, Waltham, USA) and X-ray diffraction (XRD) diffractometer (D8 Advance, Bruker, Germany).

### Membrane characterization and Cas9 protein quantification

CMs characterization were performed by sodium dodecyl sulfate–polyacrylamide gel electrophoresis (SDS-PAGE) method as previously described [40]. First, protein electrophoresis was performed using a standard protocol. Then, proteins bands were visualized by staining in Coomassie blue followed by overnight destaining in water. For western blot (WB) analysis, cell membrane proteins were electrophoretically blotted onto a nitrocellulose membrane. Membranes were probed using designated antibodies, including Na^+^/K^+^-ATPase (Proteintech, #14418-1-AP), pan-cadherin (bimake, #A5614), Histone H3 (bimake, #A5737), cytochrome c (bimake, #A5184), GAPDH (Proteintech, #60004-1-Ig), and HRP-conjugated Affinipure Goat Anti-Rabbit IgG (Proteintech, #SA00001-2). Signals were visualized by ECL reagents (Millipore, USA).

### Cell lines and animal models

HepAD38 and HepG2.2.15 cells were cultured in DMEM supplemented with 10% FBS, 100 U/ml penicillin/streptomycin and 400 μg/ml G418 and incubated at 37 °C in a 5% CO_2_ humidified atmosphere. Then, 2 μg/ml tetracycline (Tet) was added to HepAD38 cells to inhibit HBV replication. In the absence of Tet, HBV replication was induced in HepAD38 cells. Cells were validated by short tandem repeat (STR) fingerprinting (Beijing Microread Gene Technology Co., China).

HBV Transgenic (HBV-Tg) mice were kindly provided by Prof. Ning-shao Xia from the School of Public Health of Xiamen University and were maintained in the specific pathogen-free (SPF)-grade laboratory animal center of Chongqing Medical University (SCXK (YU) 2017-0001). Male 6-week-old HBV-Tg mice were used in this study.

### Controlled Cas9 RNP release by NIR laser

The Cas9 RNP release was investigated using an 808 nm wavelength NIR laser (Changchun Laser Technology Co., Ltd, China). The UV absorption spectra was determined by a Nano-500 spectrophotometer.

### In vitro biocompatibility of UCNPs-Cas9@CM

To assess the biocompatibility of UCNPs-Cas9@CM, i. Cell viability assay, ii. Hemolysis experiment and iii. Inflammatory cytokine detection was performed. Briefly, i. Cells were cultured in 96-well plates for 12 h. Then, they were incubated for 48 h under different treatments, including different concentrations of UCNPs-Cas9@CM, NIR power and irradiation time. Next, 10% CCK-8 solution was added to each well followed by 4 h of incubation. Cell viability was quantified by measuring OD450. ii. The hemolysis assay was performed to evaluate the safety of UCNPs-Cas9@CM for in vivo applications. A 2% (v/v) RBC suspension of mouse blood cells was prepared and different concentrations of UCNPs-Cas9@CM nanoparticles were added. Distilled water and PBS were used as positive and negative controls, respectively. After incubation at 37 °C for 1 h, the suspension was centrifuged and the absorbance measured at 540 nm. iii. Quantitative Real-Time-Polymerase Chain Reaction (qRT-PCR) was used to determine the mRNA expression levels of inflammatory factors (TNF-α, IL-1β and IL-8). Separately, cells were treated with PBS, UCNPs-Cas9@CM (Cas9: 0.06 μM) and LPS (50 μg/ml). After 3 h of incubation, an 808 nm NIR laser was triggered for 20 min (2 W/cm^2^, 2 min break after 1 min irradiation). After another 24 h, cellular RNA was extracted using the All Pure DNA/RNA Kit and reversed transcribed into cDNA. SYBR Green qPCR Master Mix (Bimake, China) and an ABI 7500 Real-Time PCR System (Applied Biosystems, USA) were used in this experiment. Target gene transcription was normalized to the GAPDH and the results analyzed by the 2^−∆∆CT^ method.

### Confocal imaging and flow cytometry analysis of cellular uptake

HepG2.2.15, HEK293T (human kidney cells), HeLa (human cervical adenocarcinoma epithelial), L929 (a mouse fibroblast cell line) and RAW264.7 (a murine macrophages) cells were seeded in 24-well culture plates plated with cell climbing slices a day before treatment. Cells were incubated with GFP-labeled UCNPs-Cas9 and UCNPs-Cas9@CM particles in DMEM medium. Then, they were fixed in 4% paraformaldehyde and sealed with a fluorescence quenching sealing tablet containing 4',6-diamidino-2-phenylindole (DAPI, Beyotime, China). Confocal Laser Scanning Microscopy (CLSM, Leica TCS SP8, Germany) was performed to observe the cells. Cellular accumulation levels of Cas9-GFP were evaluated by flow cytometry (Beckman Coulter, USA).

### Isolation and quantification of HBV DNA, HBV cccDNA and HBV 3.5 kb mRNA

HBV DNA was isolated from Hep2.2.15 and HepAD38 cells as previously described [52]. Briefly, cells were lysed in 0.5 ml core lysis buffer (1 mM EDTA, 10 mM Tris-HCl, 1% NP-40, 2% sucrose) at 37 ºC for 15 min. After centrifugation, the resulting supernatant was treated with 4 μl DNase I (5 IU/mL) and 5 μl MgCl_2_ (1 M) for 4 h at 37 °C. The mixture was further centrifuged and incubated with 200 μl of 35% PEG8000 for 1 h in an ice bath. Then, proteinase K was used to release viral DNA at 45 ºC for 12 h. HBV DNA were purified by phenol/chloroform (1:1) extraction, precipitated with ethanol and resuspended in a TE buffer. The extracted HBV DNA was quantified with a pair of specific primers (HBV-F_2150_ and HBV-R_2300_) using the absolute qPCR method.

HBV cccDNA was isolated by a modified Hirt method. Cells were lysed in 0.5 ml SDS lysis buffer (10 mM EDTA, 50 mM Tris-HCl, 150 mM NaCl, 1% SDS) at 37 ºC for 15 min, after which they were mixed with 125 μl KCl (2.5 M) and stored at 4 °C overnight. After centrifugation, the resulting supernatant was extracted by phenol/chloroform (1:1), precipitated with isopropanol, purified with ethanol and resuspended in TE buffer. HBV cccDNA was detected by Taq-man probe qRT-PCR. Extraction and analysis methods for total cellular RNA were as described above.

### Assessments of HBsAg and HBeAg levels in in vitro experiments

HBsAg and HBeAg levels from HepG2.2.15 and HepAD38 cell culture supernatants were assayed by a commercial ELISA kit (Kehua Biotech, Shanghai, China).

### In vivo biocompatibility and pharmacokinetic properties of UCNPs-Cas9@CM

To assess the toxicity of NIR-controlled UCNPs-Cas9@CM in vivo, HBV-Tg mice were randomized into 3 groups: PBS + NIR (negative control, n = 3), UCNPs-Cas9 + NIR (n = 3), UCNPs-Cas9@CM + NIR (n = 3). Preparations were injected by tail vein injection, and mice livers were irradiated with 808 nm NIR laser irradiation for 30 min (2 W/cm^2^, 2 min break after 1 min irradiation). This phase was continued for 3 days, which meant that 100 μl of UCNPs-Cas9@CM or UCNPs-Cas9 (40 μg Cas9) solution were injected everyday followed by 30 min of NIR irradiation. For all the experiments in HBV-Tg mice, final concentrations of UCNPs-Cas9@CM were twofold those of cell experiments. Following 14 days of these treatment, mice blood was collected by orbital bleeding for blood biochemistry and blood routine examination, after which the hearts, livers, spleens, lungs and kidneys were harvested for paraffin sectioning and hematoxylin-eosin (H&E) assays histopathological evaluations.

For the excretion study, urine and excrements for mice from the experimental groups were collected every 24 h for a total of 7 times using metabolic cages. Samples were evaluated by inductively coupled plasma mass spectrometry (ICP-MS).

### Analysis of HBsAg, HBeAg and HBV DNA in HBV-Tg mice

HBsAg and HBeAg levels in mice serum were quantified using a Hepatitis B Virus Antigen Assay Kit (Chemiluminescence analysis, CLIA) and a I3000 Automatic Chemiluminescence Immunoassay Analyzer (Maccura, China). The HBV DNA detection in mice serum was performed using a Hepatitis B Viral DNA Quantitative Fluorescence Diagnostic Kit (Sansure Biotech Inc., China).

### Immunohistochemistry (IHC)

Mice liver tissues were fixed in 10% formalin and paraffin-embedded. They were assayed by anti-HBsAg (MXB Biotechnologies, #MAB-0847) and anti-HBcAg (MXB Biotechnologies, #RAB-0090). Counterstaining with secondary anti-rabbit/mouse IgG and visualized using a 3, 3’-diaminobenzidine (DAB) substrate by a DAB Detection Kit (Polymer) (MXB Biotechnologies, #Kit-0014).

### T7E1 and DNA sequencing analysis

Genomic DNA was extracted using the AllPure DNA/RNA Kit (Magen, China). DNA fragments, including nuclease target sites were amplified by PCR using PrimeSTAR (TaKaRa, China) and purified using a Qiagen Purification Kit (Hilden, Germany). The product was digested with T7E1 and analyzed by agarose gel electrophoresis. To analyze DNA sequences, the amplified DNA was cloned into a pTOPO-Blunt Vector (Mei5, China) followed by Sanger sequencing.

### Off-target gene-editing analysis

Potential off-target sites for sgRNA were predicted using a bioinformatics software. Genomic DNA, as a template for PCR, was extracted from human cells and mice liver tissues and their off-target genomic sites amplified by specific primer pairs. Amplified DNAs were digested by T7E1 and analyzed by agarose gel electrophoresis.

### Statistical analysis

Data are presented as mean ± standard deviation (SD). Comparisons of means between and among groups were performed by the Student’s t-test and one-way analysis of variance (ANOVA). *p* ≤ 0.05 was considered significant.

## Supplementary Information

**Additional file 1: Fig. S1**. Characterization of UCNPs.** Fig. S2**. Full length PCR fragment (FL) with sgRNA target sequence was incubated with the Cas9/sgRNA complexes respectively, and Cas9/sgRNA17 showed a higher cleavage efficiency. **Fig. S3**. Characterization of CMs. **Fig. S4**. NIR-responsive negative groups in response to Cas9 release. **Fig. S5**. Cells with internalized UCNPs-Cas9@CM were observed by CLSM. **Fig. S6**. Z-stack and z-stack 3D images of the cells that incubated with UCNPs-Cas9@CM at 6 h with/without NIR irradiation. **Fig. S7**. Immune escape study of UCNPs-Cas9@CM. **Fig. S8**. Study on the cytotoxicity of UCNPs-Cas9@CM. **Fig. S9**. Homotypic target study of UCNPs-Cas9@CM. **Fig. S10**. The temperature profiles of UCNPs-Cas9@CM solution after NIR(+/-) irradiation. **Fig. S11**. Cell viability tests. **Fig. S12**. Sanger sequencing profiles of the Indel DNA. **Fig. S13**. Off-target effects of UCNPs-Cas9@CM in vitro. **Fig. S14**. The homing capability of UCNPs-Cas9@CM in vivo. **Fig. S15**. The biocompatibility and toxicity analysis of UCNPs-Cas9@CM in vivo. **Fig. S16**. Off-target effects of UCNPs-Cas9@CM in vivo. **Table S1**. Sequences of DNA oligos.

## Data Availability

All data analyzed during this study are included in this published article.

## Abbreviations

HBV: Hepatitis B virus
IFNs: Interferons
NUCs: Nucleoside analogs
cccDNA: Covalently closed circular DNA
CHB: Chronic hepatitis B
CRISPR: Clustered Regularly Inter Spaced Palindromic Repeats
CRISPR/Cas9: CRISPR-associated protein 9
sgRNA: Single guide RNA
AAV: Asadeno-Associated Virus
Cas9 RNP: Cas9 ribonucleoprotein
CPPs: Cell-penetrating peptides
NIR: Near-infrared
UCNPs: Lanthanide upconversion nanoparticles
CMs: Cell-membrane-derived vesicles
PCB: PC Biotin-NHS Ester
NLS: Nuclear localization sequence
OA: Oleic acid
ODE: 1-Octadecene
PAA: Poly (acrylic acid)
NHS: N-hydroxysuccinimide
EDC: N-(3dimethylaminopropyl)-N’-ethylcarbodiimide hydrochloride
CCK-8: Cell counting kit-8
PMSF: Phenylmethanesulfonyl fluoride
LPS: Lipopolysaccharide
T7E1: T7 endonuclease 1
FBS: Fetal bovine serum
RT: Room temperature
TEM: Transmission electron microscopy
SEM: Scanning electron microscopy
FTIR: Fourier Transform infrared spectroscopy
XRD: X-ray diffraction
SDS-PAGE: Sodium dodecyl sulfate polyacrylamide gel electrophoresis
WB: Western blot
Tet: Tetracycline
STR: Short tandem repeat
HBV-Tg: HBV Transgenic
SPF: Specific pathogen-free
qRT-PCR: Quantitative Real-Time-Polymerase Chain Reaction
DAPI: 4′,6-Diamidino-2-phenylindole
CLSM: Confocal Laser Scanning Microscopy
H&E: Hematoxylin–eosin
IHC: Immunohistochemistry
CLIA: Chemiluminescence Immunoassay
ICP-MS: Inductively coupled plasma mass spectrometry
DAB: 3, 3’-Diaminobenzidine
HRTEM: High Resolution TEM
SAED: Selected area electron diffraction
MFI: Mean fluorescence intensity
WT: Wild-type; Mut: mutation
