# Supplementary material for: CRISPR/Cas9 delivery by NIR-responsive biomimetic nanoparticles for targeted HBV therapy
Source: J Nanobiotechnology. 2022 Jan 6;20:27. doi: 10.1186/s12951-021-01233-4 (PMC8740473; doi:10.1186/s12951-021-01233-4)
Supplement: Supplementary file 1 — Additional file 1: Fig. S1. Characterization of UCNPs. Fig. S2. Full length PCR fragment (FL) with sgRNA target sequence was incubated with the Cas9/sgRNA complexes respectively, and Cas9/sgRNA17 showed a higher cleavage efficiency. Fig. S3. Characterization of CMs. Fig. S4. NIR-responsive negative groups in response to Cas9 release. Fig. S5. Cells with internalized UCNPs-Cas9@CM were observed by CLSM. Fig. S6. Z-stack and z-stack 3D images of the cells that incubated with UCNPs-Cas9@CM at 6 h with/without NIR irradiation. Fig. S7. Immune escape study of UCNPs-Cas9@CM. Fig. S8. Study on the cytotoxicity of UCNPs-Cas9@CM. Fig. S9. Homotypic target study of UCNPs-Cas9@CM. Fig. S10. The temperature profiles of UCNPs-Cas9@CM solution after NIR(+/-) irradiation. Fig. S11. Cell viability tests. Fig. S12. Sanger sequencing profiles of the Indel DNA. Fig. S13. Off-target effects of UCNPs-Cas9@CM in vitro. Fig. S14. The homing capability of UCNPs-Cas9@CM in vivo. Fig. S15. The biocompatibility and toxicity analysis of UCNPs-Cas9@CM in vivo. Fig. S16. Off-target effects of UCNPs-Cas9@CM in vivo. Table S1. Sequences of DNA oligos. [file 12951_2021_1233_MOESM1_ESM.docx]

**Supporting information**

**CRISPR/Cas9 delivery by** [**NIR-responsive biomimetic nanoparticles for targeted HBV therapy**](https://pubmed.ncbi.nlm.nih.gov/34522579)

Dan Wang^1, 2#^, Lin Chen^1#^, Chengbi Li^2#^, Quanxin Long^1^, Qing Yang^2^, Ailong Huang^1^, Hua Tang^1*^

^1^Key Laboratory of Molecular Biology for Infectious Diseases (Ministry of Education), Institute for Viral Hepatitis, Department of Infectious Diseases, The Second Affiliated Hospital, Chongqing Medical University, Chongqing 400016, China.

^2^ The People's Hospital of Rongchang District, Chongqing 402460, China.

**^#^** Dan Wang, Lin Chen and Chengbi Li equally contributed to this work

******* Corresponding author: Hua Tang

1 Yi Xue Yuan Road, Chongqing, 400016, China

Tel: +86 23 68486780. Fax: +86 23 68486780.

E-mail: [tanghua86162003@cqmu.edu.cn](mailto:tanghua86162003@cqmu.edu.cn)


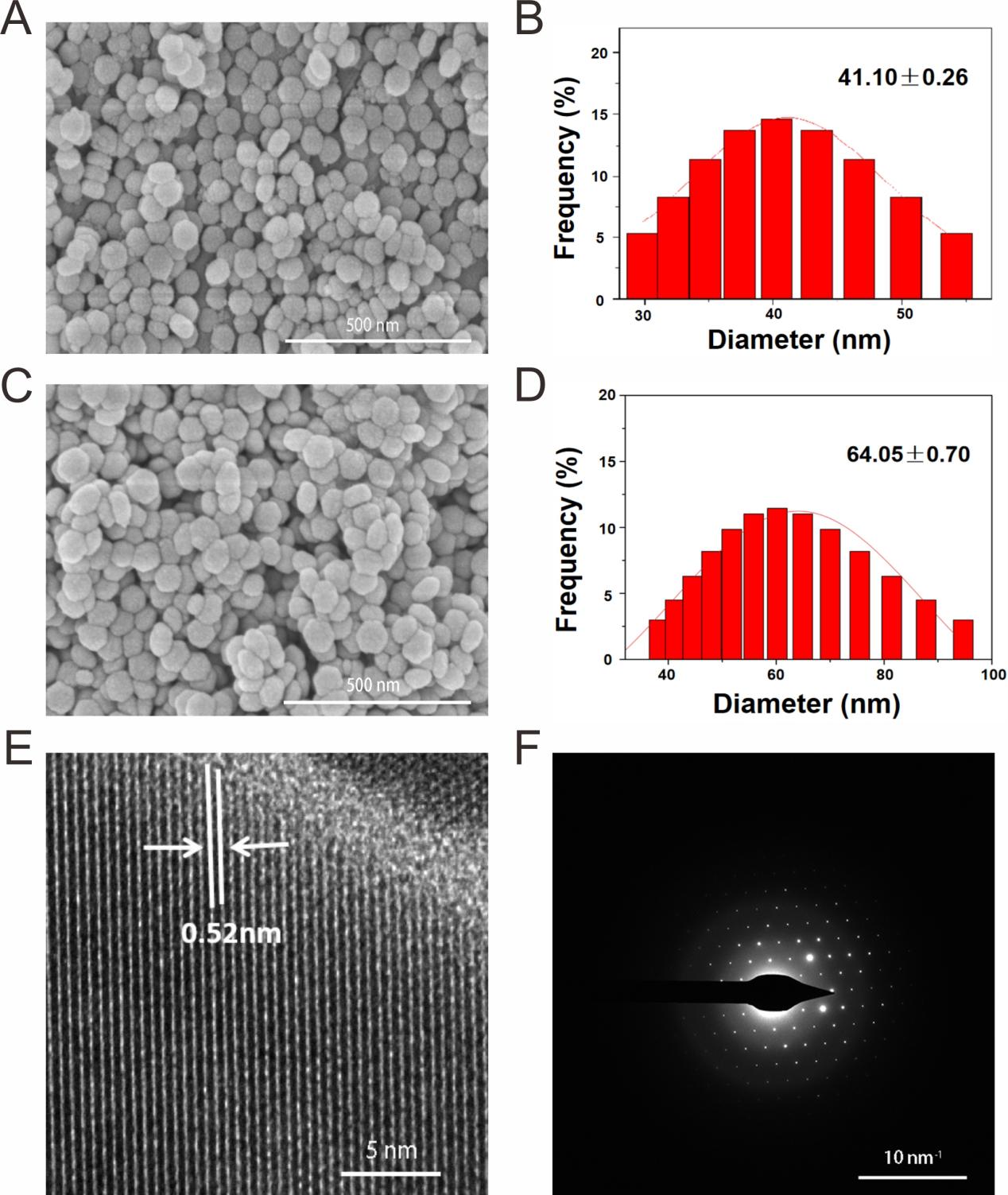


**Fig. S1** Characterization of UCNPs. SEM images **(A)** and size histograms **(B)** of NaYF_4_:Yb/Tm/Ca. SEM images **(C)** and size histograms **(D)** of NaYF_4_:Yb/Tm/Ca@NaYF_4_:Yb/Nd. **(E)** HRTEM images and **(F)** SAED of NaYF_4_:Yb/Tm/Ca@NaYF_4_:Yb/Nd.


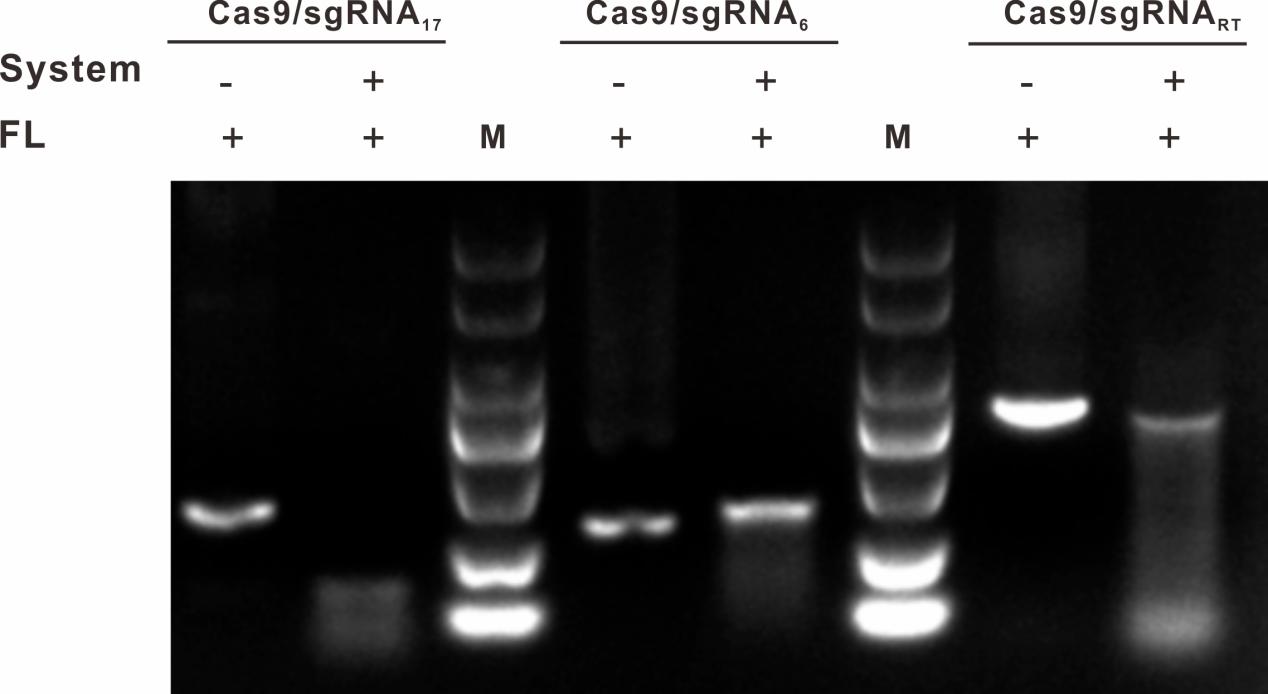


**Fig. S2** Full length PCR fragment (FL) with sgRNA target sequence was incubated with the Cas9/sgRNA complexes respectively, and Cas9/sgRNA_17_ showed a higher cleavage efficiency.


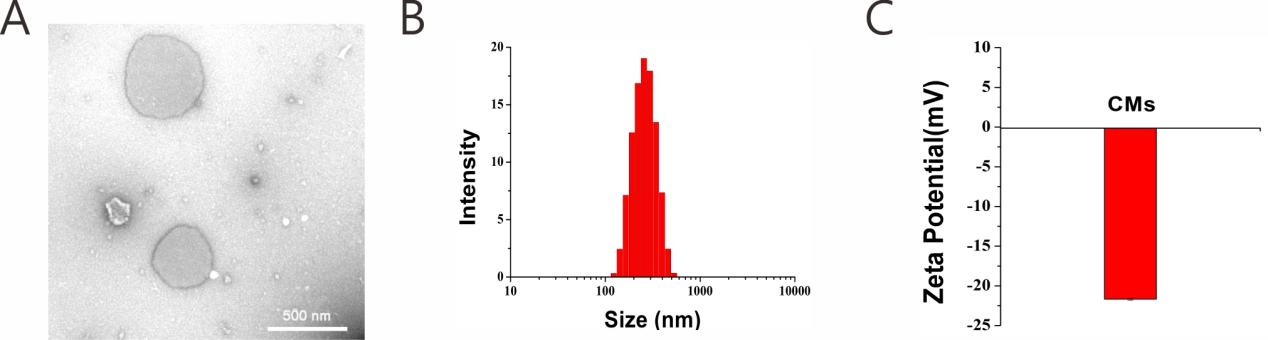


**Fig. S3** Characterization of CMs. TEM images **(A)**, Hydrodynamic diameters **(B)** and zeta potential **(C)** of CMs.


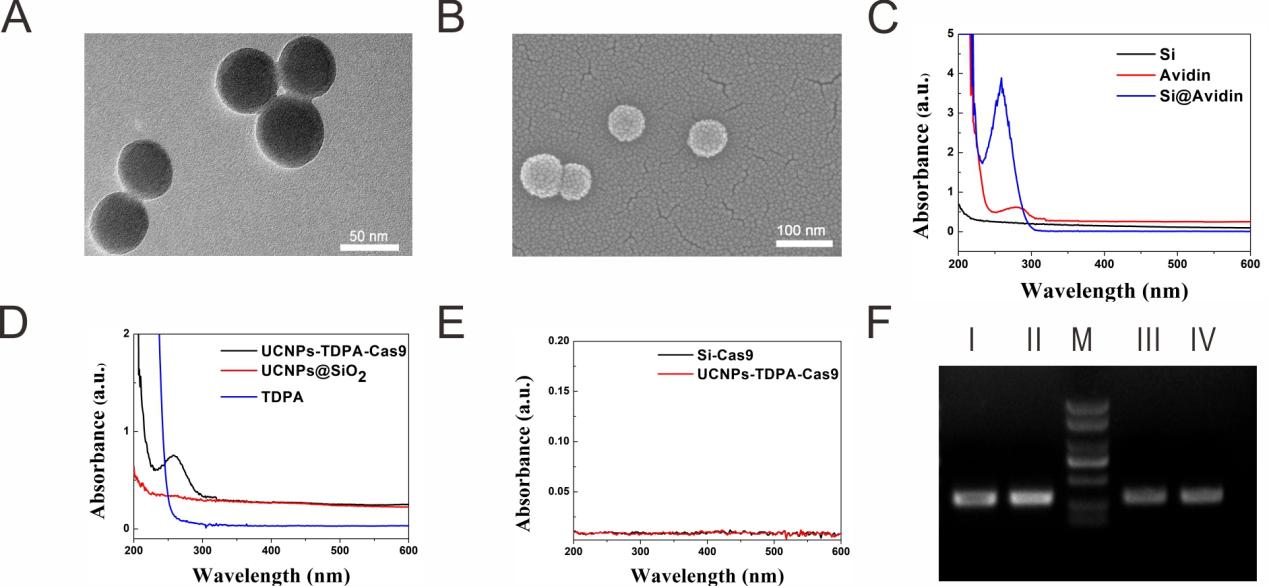


**Fig. S4** NIR-responsive negative groups in response to Cas9 release. TEM **(A)** and SEM **(B)** images of Si. **(C)** UV-vis absorption spectrum of Si, Avidin and Si@Avidin. **(D)** UV-vis absorption spectrum of TDPA, UCNPs@SiO2 and UCNPs-TDPA-Cas9. **(E)** UV-vis absorption spectra of the supernatant solution of Si-Cas9 and UCNPs-TDPA-Cas9 upon NIR irradiation (NIR power: 2.0 W/cm^2^, fixed 20 min). **(F)** Cas9 protein verification. Si-Cas9 group: I. FL; II. FL+sgRNA+ the supernatant. UCNPs-TDPA-Cas9 group: III. FL; IV. FL+sgRNA+ the supernatant. FL: Full length PCR fragment with sgRNA target sequence.


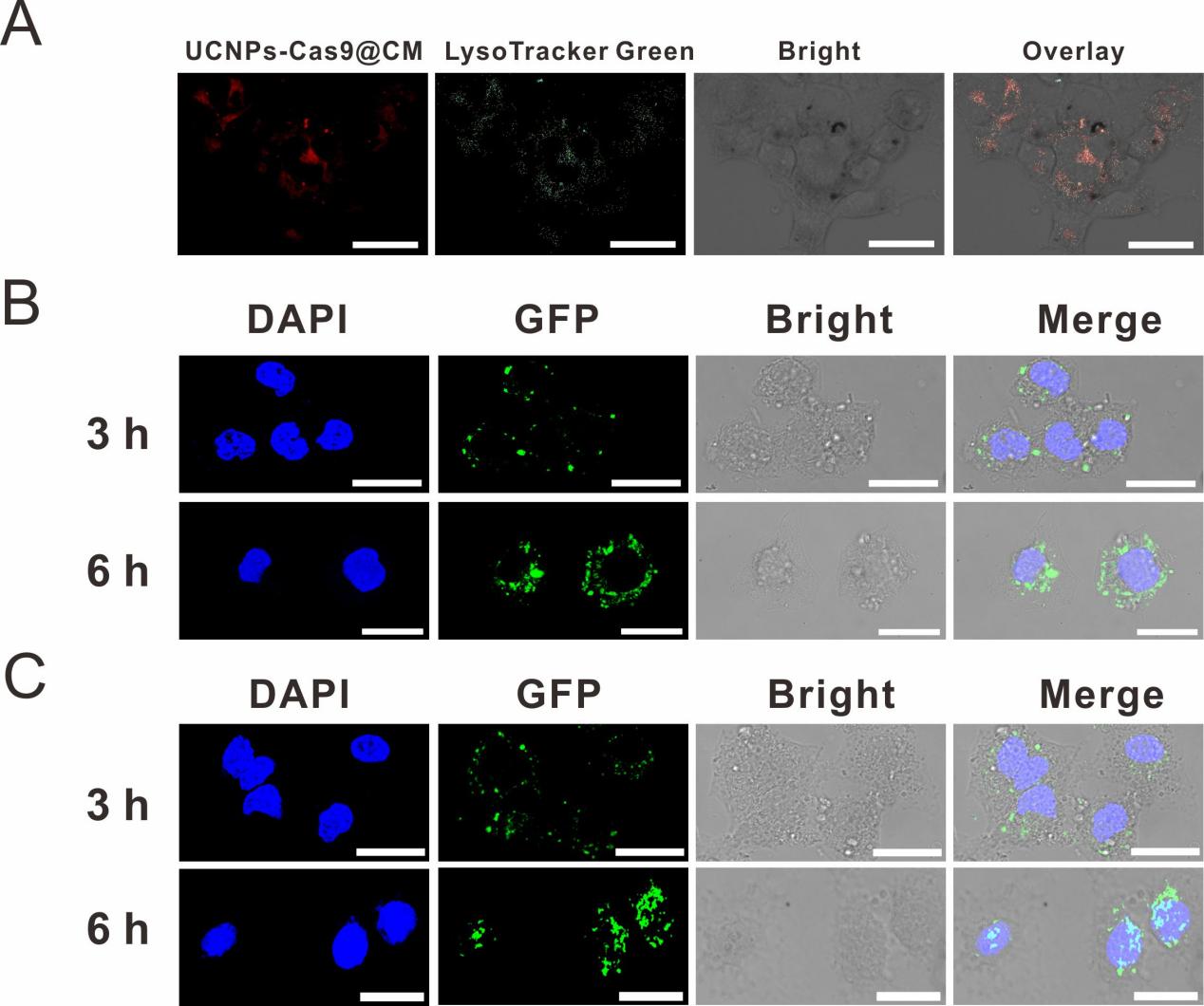


**Fig. S5** Cells with internalized UCNPs-Cas9@CM were observed by CLSM. **(A)** CLSM images of HepG2.2.15 cells, which were treated with UCNPs-Cas9@CM for 3 h and stained with LysoTracker Green. NLS-Cas9-NLS protein was used in this experiment. **(B)** Cells were co-culture with UCNPs-Cas9@CM for 3 h and 6 h, respectively, and no green color was observed in cell nuclei. **(C)** Cells were incubated with UCNPs-Cas9@CM under NIR irradiation. 6 h later, Cas9 was observed in cell nuclei (green: GFP-labeled Cas9; blue: DAPI). NLS-Cas9-EGFP protein was used in this experiment. Scale bars: 25 μm.


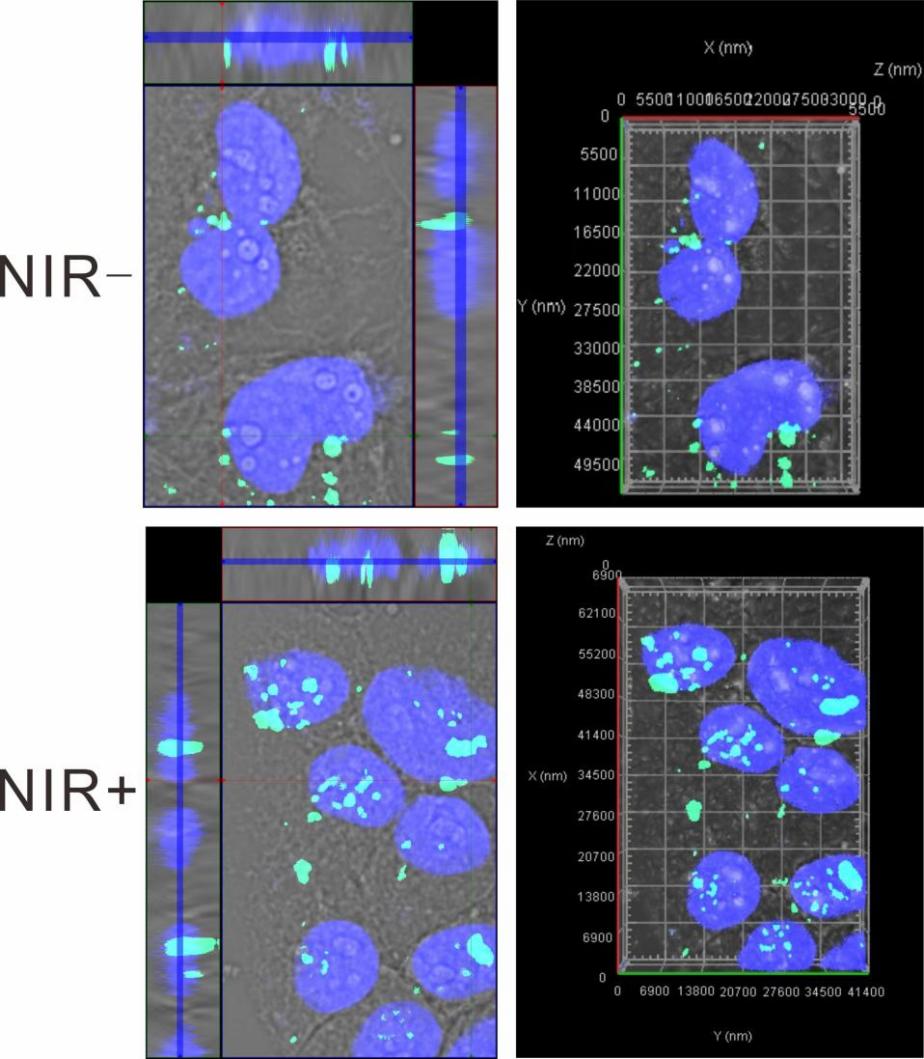


**Fig. S6** Z-stack and z-stack 3D images of the cells that incubated with UCNPs-Cas9@CM at 6 h with/without NIR irradiation (green: GFP-labeled Cas9; blue: DAPI).


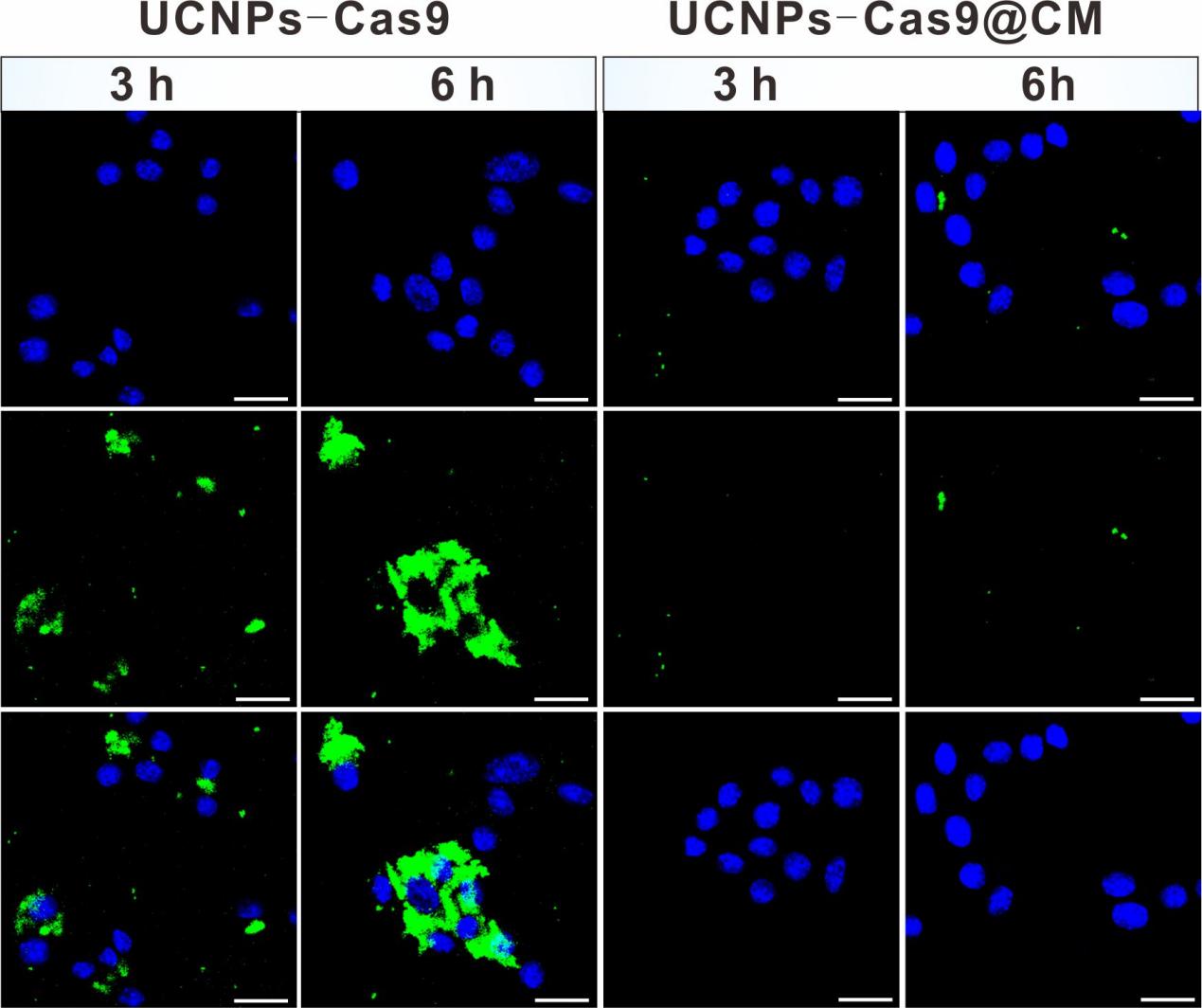


**Fig. S7** Immune escape study of UCNPs-Cas9@CM. CLSM images of RAW264.7 cells upon 3 h or 6 h of co-culture with UCNPs-Cas9 and UCNPs-Cas9@CM. Scale bars: 25 μm.


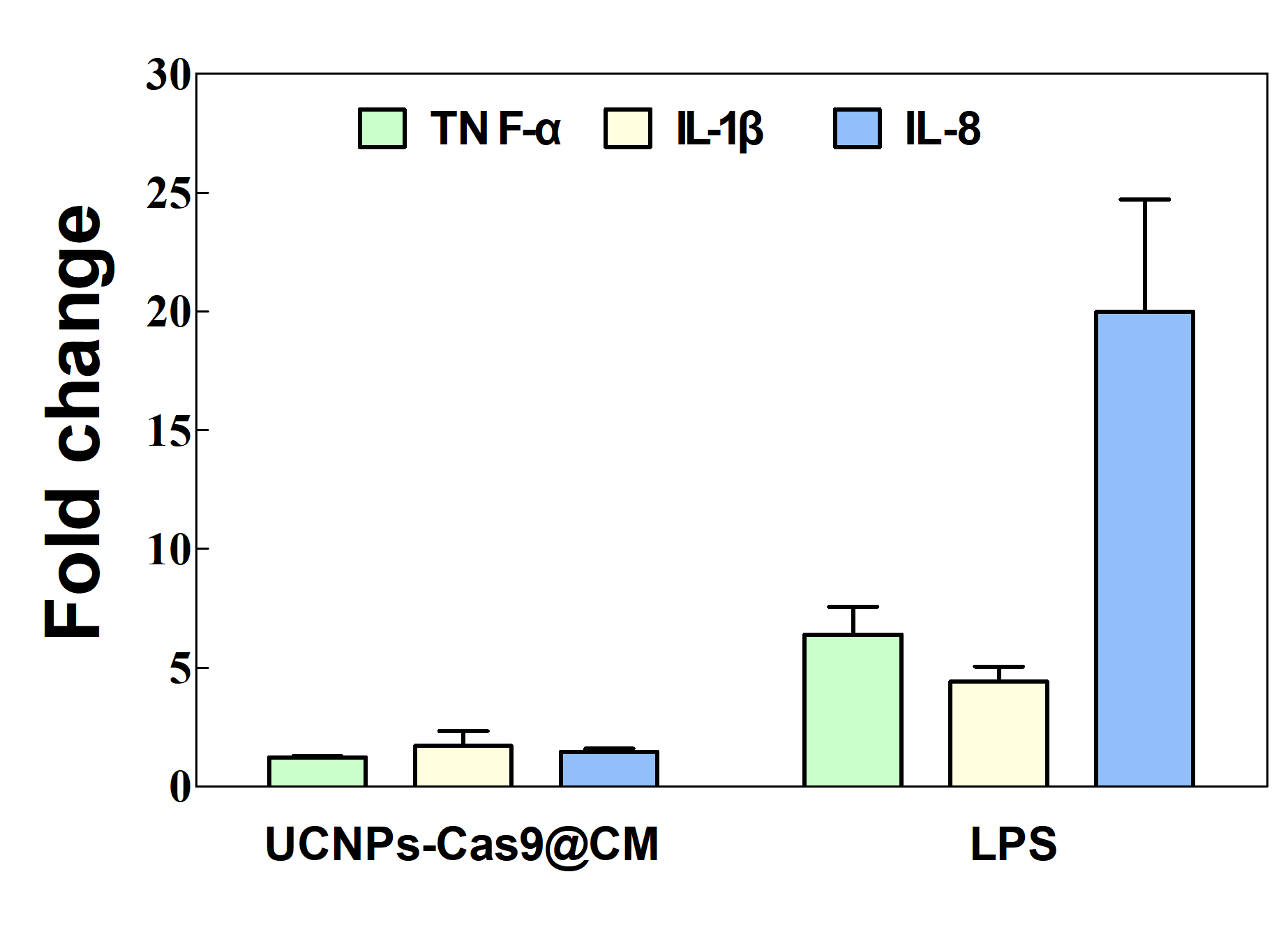


**Fig. S8** Study on the cytotoxicity of UCNPs-Cas9@CM. The mRNA expression levels of inflammatory cytokines (TNF-α, IL-1β, IL-8) of UCNPs-Cas9@CM groups and LPS groups relative to PBS. Data are shown as mean±SD (n = 3).

**
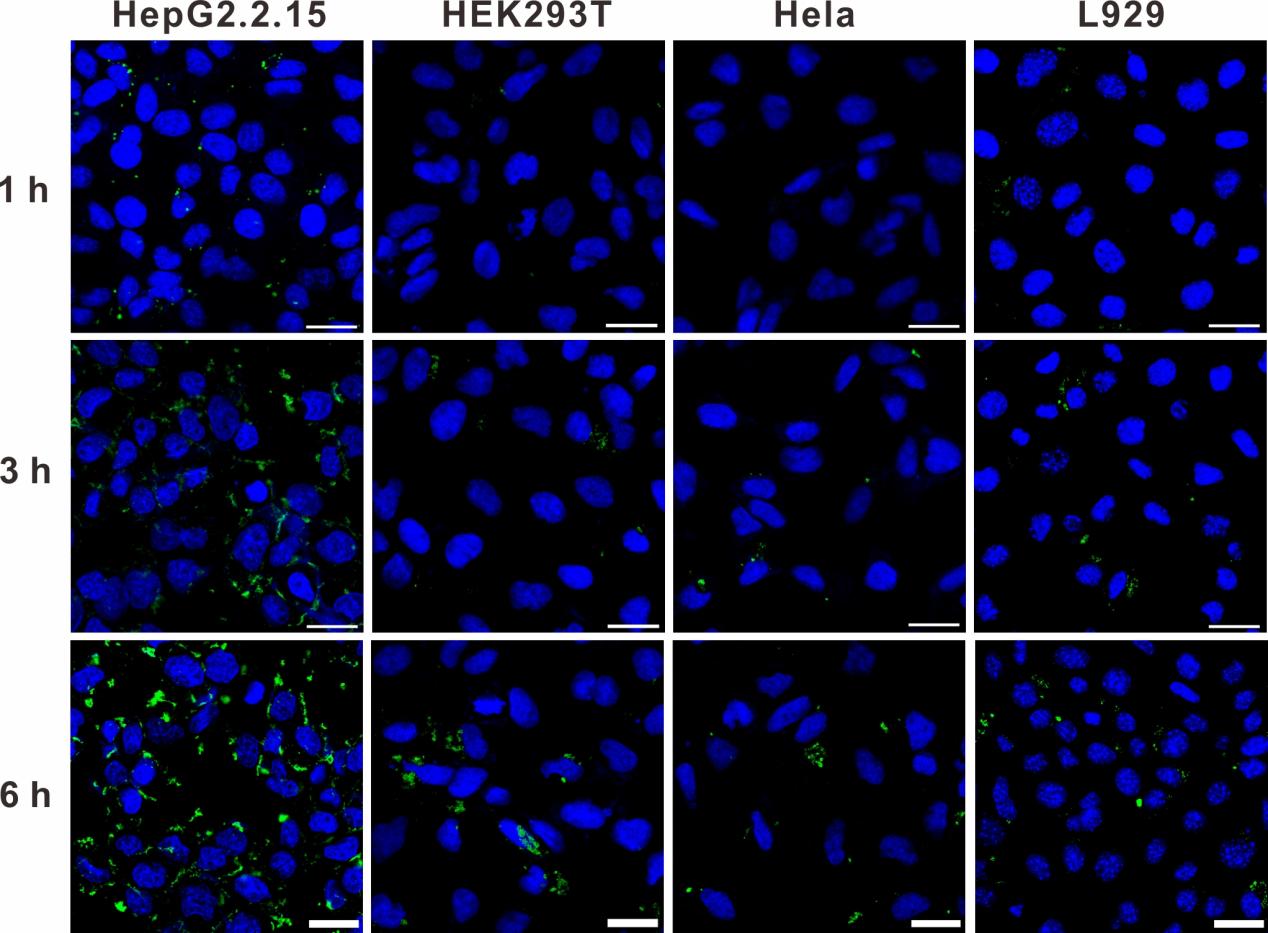
**

**Fig. S9** Homotypic target study of UCNPs-Cas9@CM. CLSM images of four cell lines upon 1 h, 3 h or 6 h of co-culture with UCNPs-Cas9@CM. Scale bars: 25 μm.


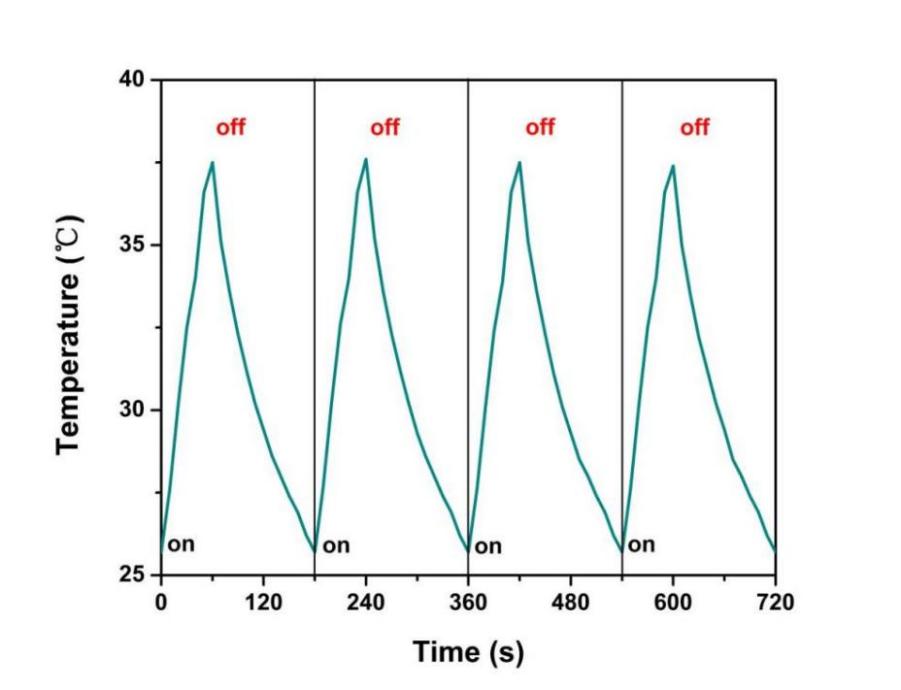


**Fig. S10** The temperature profiles of UCNPs-Cas9@CM solution after NIR(+/-) irradiation.


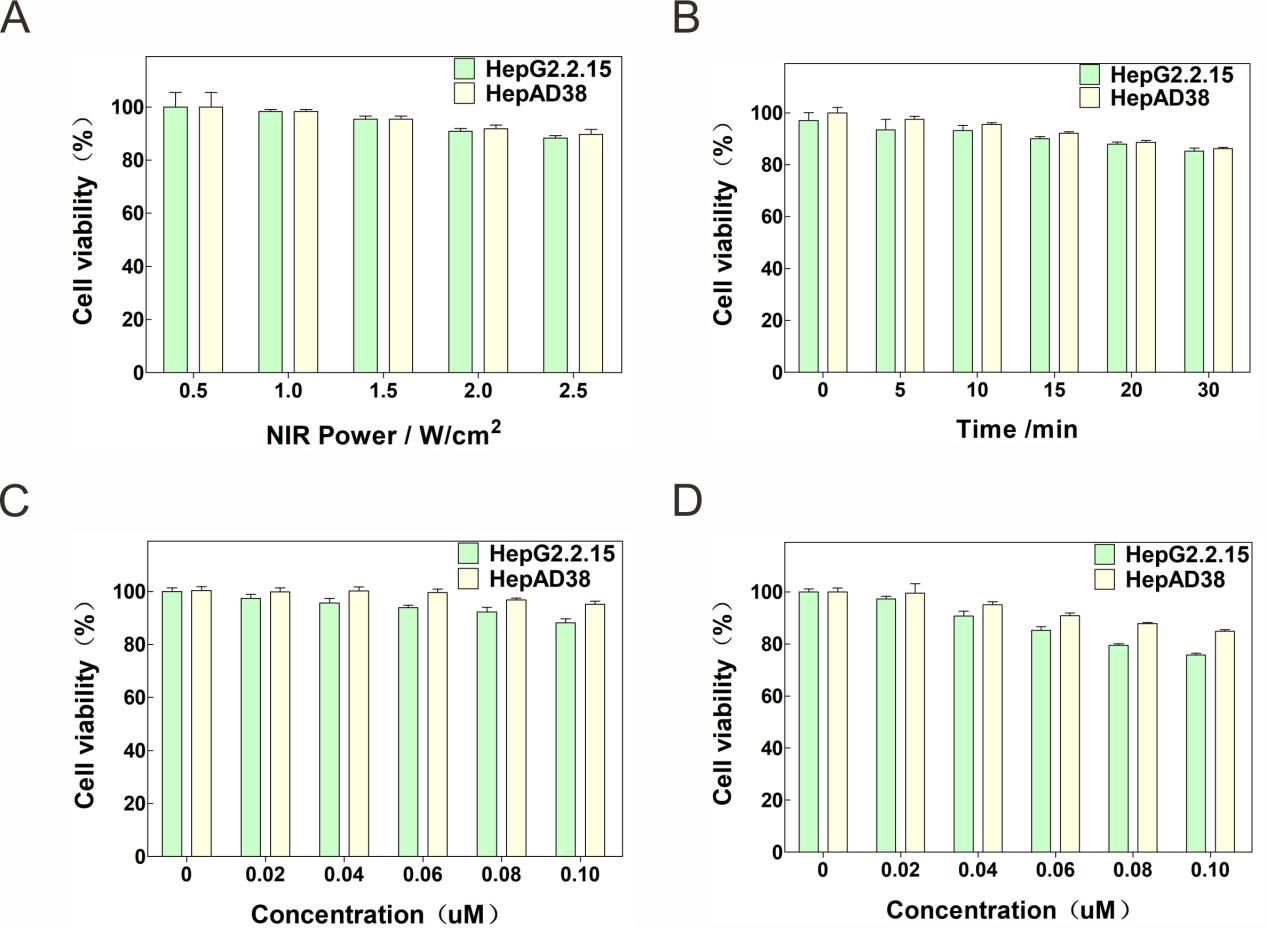


**Fig. S11** Cell viability tests. **(A)** Different NIR power (fixed 20 min, no UCNPs-Cas9@CM), **(B)** Different irradiation time (NIR power: 2.0 W/cm^2^, no UCNPs-Cas9@CM), **(C)** Different concentration of UCNPs-Cas9@CM (no NIR) and **(D)** Different concentration of UCNPs-Cas9@CM (NIR power: 2.0 W/cm^2^, fixed 20 min). The UCNPs-Cas9@CM concentration is labeled in forms of Cas9 concentration (μM).

**
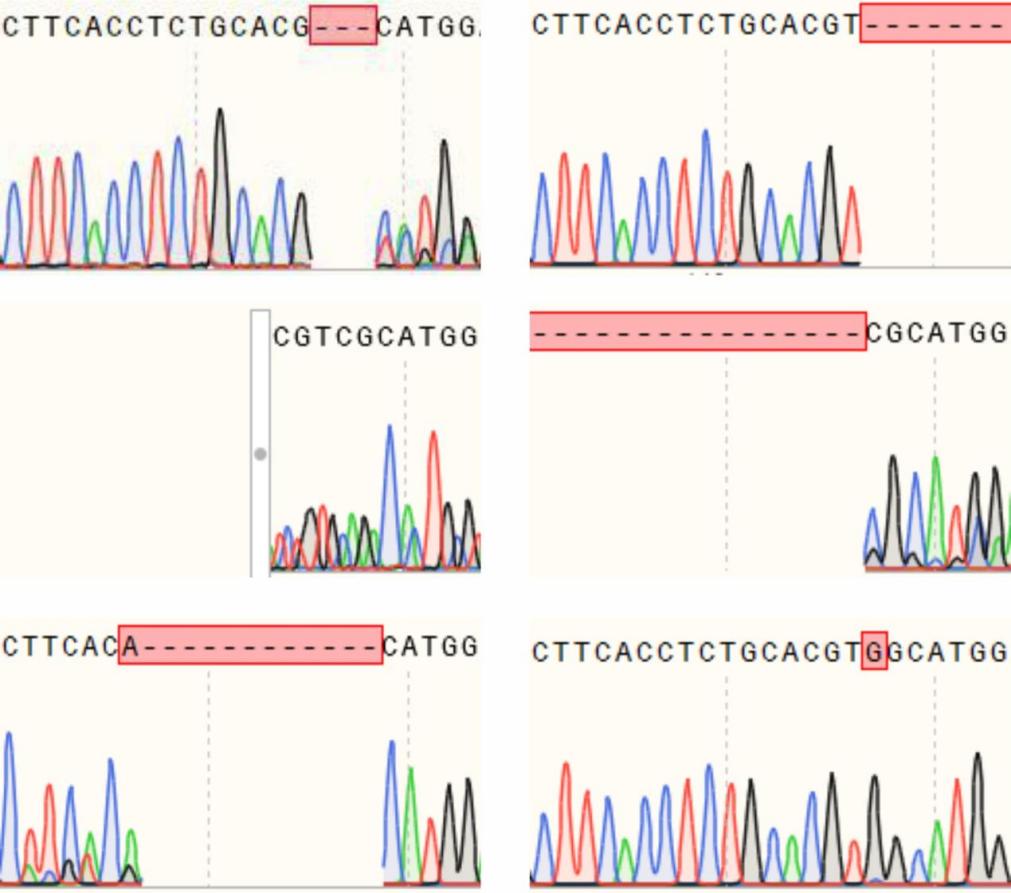
**

**Fig. S12** Sanger sequencing profiles of the indel DNA.


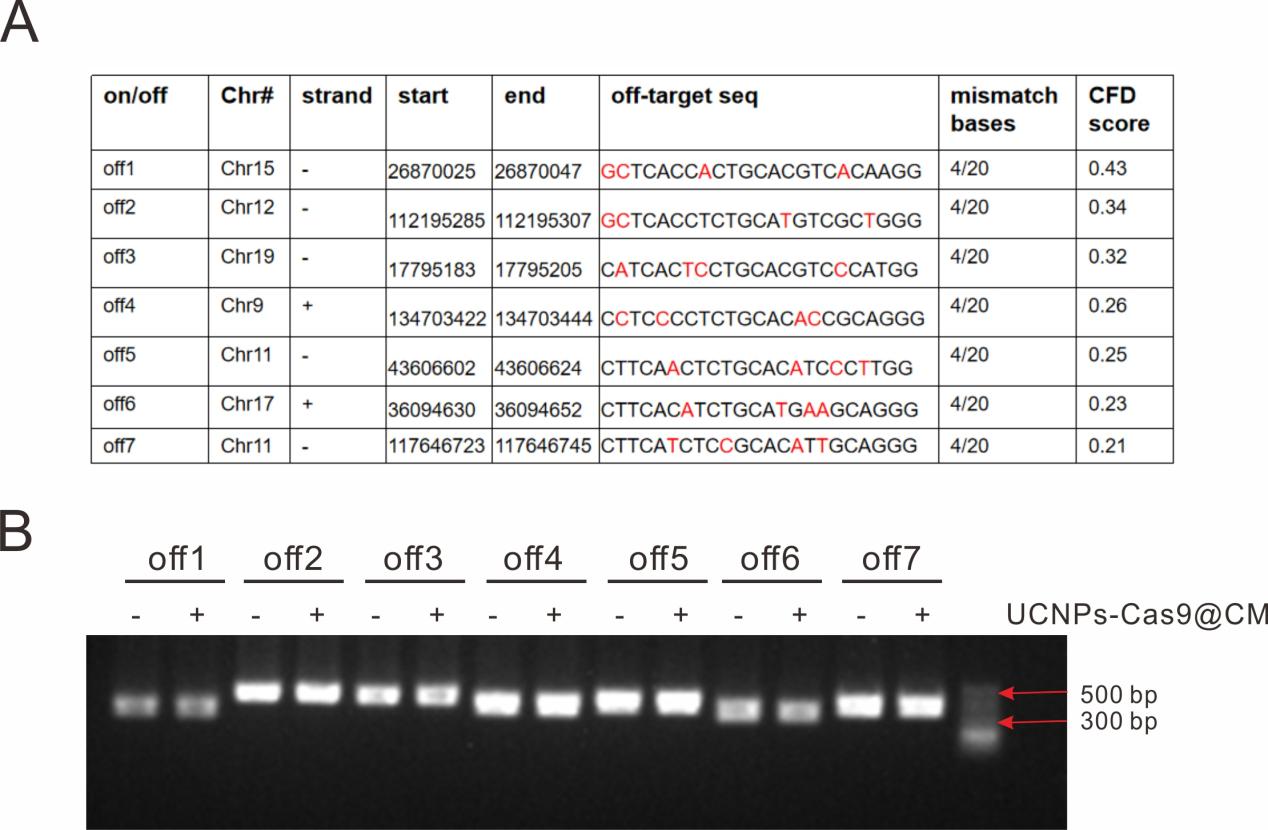


**Fig. S13** Off-target effects of UCNPs-Cas9@CM *in vitro*. **(A)** List of off-target sequences with mismatch sites and mismatched bases shown in red. **(B)** T7E1 assay was performed to identified the potential off-target effects in various target sequence of chromosomes (Chr#).


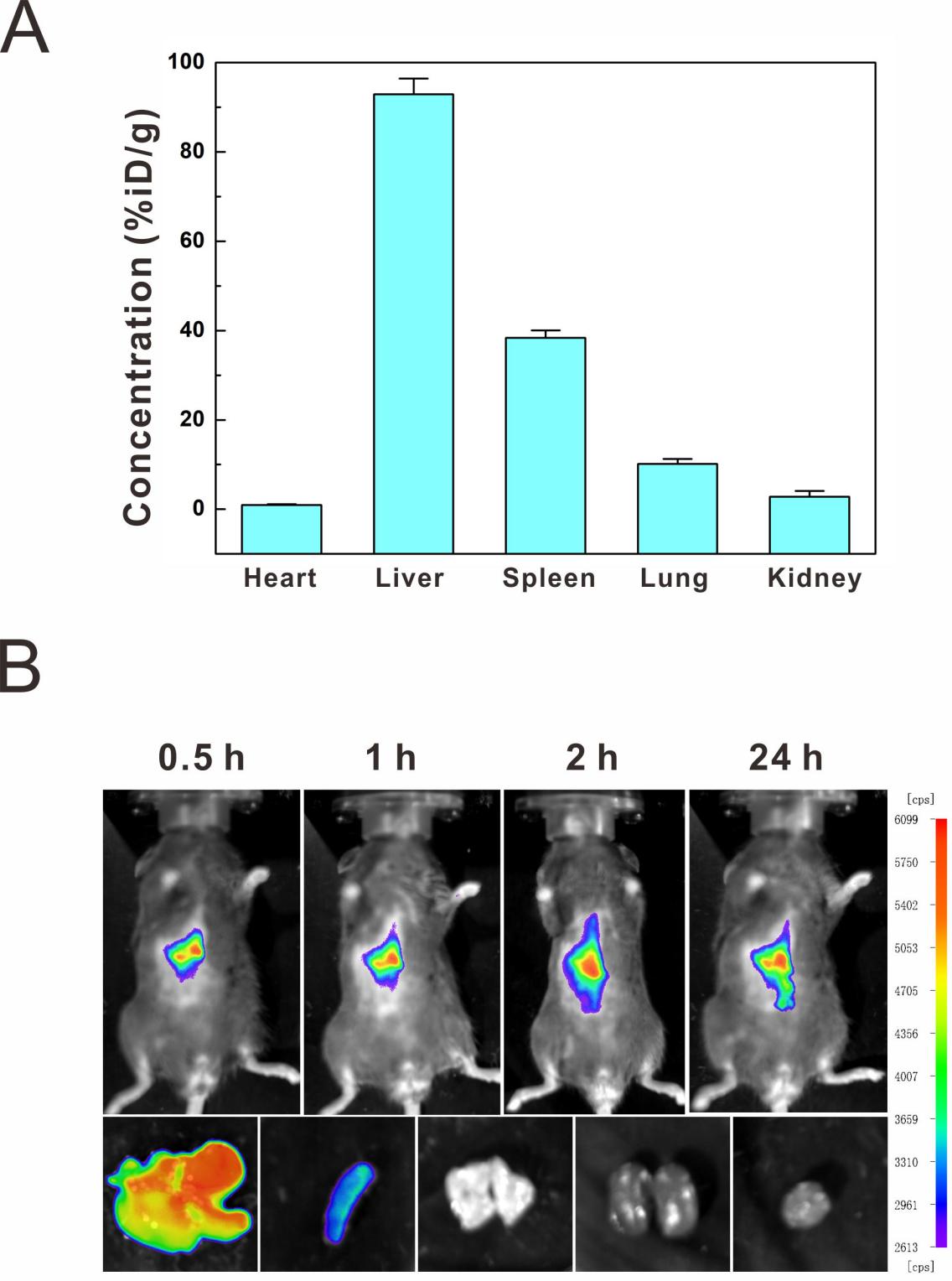


**Fig. S14** The homing capability of UCNPs-Cas9@CM *in vivo*. **(A)** The lanthanide ion (Y^3+^) content of major organs from the mice. **(B)** UCNPs-Cas9@CM were stained with DiR and injected via tail vein. Liver dominated localization of the CMs-functionalized UCNPs-Cas9@CM as revealed by bioluminescence imaging.


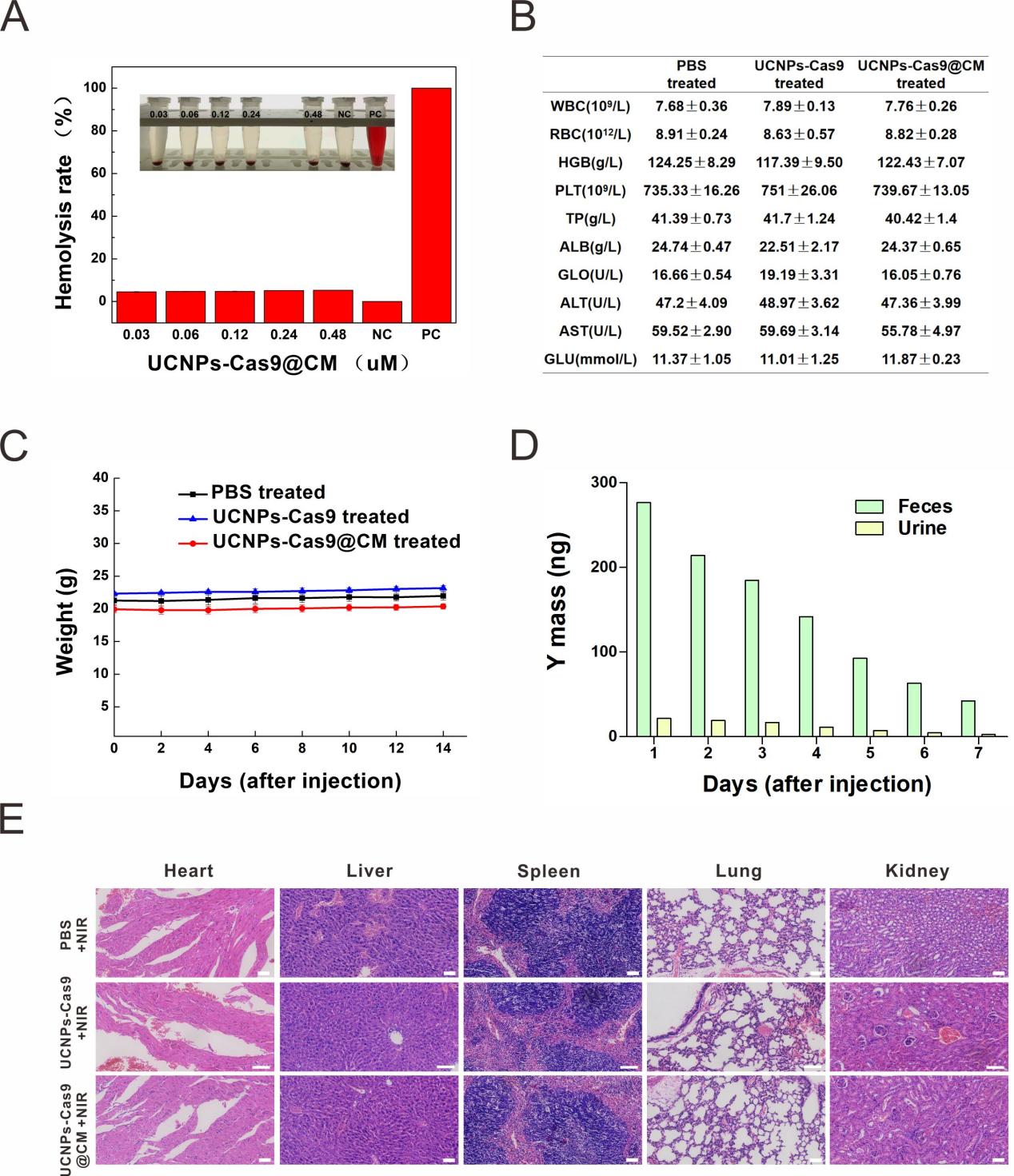


**Fig. S15** The biocompatibility and toxicity analysis of UCNPs-Cas9@CM *in vivo*. **(A)** Hemolysis detection of UCNPs-Cas9@CM at various concentrations. The UCNPs-Cas9@CM concentration was labeled in forms of Cas9 concentration (uM). **(B)** The blood routine and blood biochemistry levels of the mice after 14 days of different treatments. **(C)** Weight changes of the mice after 14 days of different treatments. **(D)** The lanthanide ion (Y^3+^) content in both feces and urine in 7 days after intravenous injection. **(E)** H&E staining of major organs from the mice after 14 days of different treatments. Scale bars: 60 μm


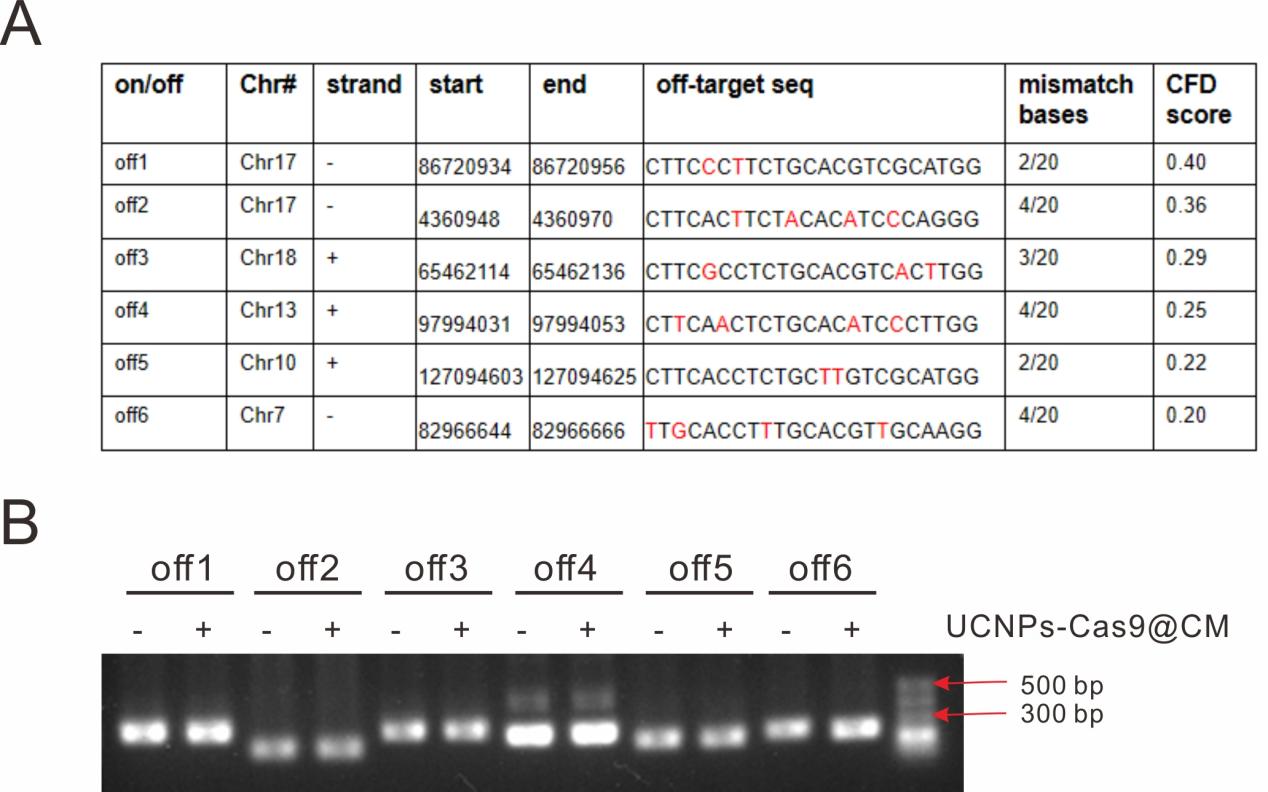


**Fig. S16** Off-target effects of UCNPs-Cas9@CM *in vivo*. **(A)** List of off-target sequences with mismatch sites and mismatched bases shown in red. **(B)** T7E1 assay was performed to identified the potential off-target effects in various target sequence of chromosomes (Chr#).

| **Table S1. Sequences of DNA oligos** | | | | |
| --- | --- | --- | --- | --- |
|  | Name | | Sequences (5'-3') | Notes |
| sgRNA Transcription  Template | sgRNA RT | | AAGCTAATACGACTCACTATAGGTTCAGTTATATGGATGATGGTTTTAGAGCTAGAAATAGCAAGTTAAAATAAGGCTAGTCCGTTATCAACTTGAAAAAGTGGCACCGAGTCGGTGCTTTTTT | T7 promoter  Target  Sequence  sgRNA  Scaffold |
|  | sgRNA 6 | | AAGCTAATACGACTCACTATAGGAATGTCAACGACCGACCTTGGTTTTAGAGCTAGAAATAGCAAGTTAAAATAAGGCTAGTCCGTTATCAACTTGAAAAAGTGGCACCGAGTCGGTGCTTTTTT |  |
|  | sgRNA 17 | | AAGCTAATACGACTCACTATAGGCTTCACCTCTGCACGTCGCAGTTTTAGAGCTAGAAATAGCAAGTTAAAATAAGGCTAGTCCGTTATCAACTTGAAAAAGTGGCACCGAGTCGGTGCTTTTTT |  |
| Primers for  T7E1 Assay | T7E1-RT | F | TTCCTCTTCATCCTGCTGCT |  |
|  |  | R | TGTAAAAGGGGCAGCAAAAC |  |
|  | T7E1-6 | F | CTCTCTTTACGCGGACTC |  |
|  |  | R | GCGCAGACCAATTTATGCC |  |
|  | T7E1-17 | F | GTCTGCCGTTCCGACCGACC |  |
|  |  | R | TGCATGGTGCTGGTGCGCAG |  |
| Real-Time PCR  Primers | mGAPDH | F | AGGTCGGTGTGAACGGATTTG |  |
|  |  | R | TGTAGACCATGTAGTTGAGGTCA |  |
|  | TNF-α | F | TCCCCAAAGGGATGAGAAGTT |  |
|  |  | R | GAGGAGGTTGACTTTCTCCTGG |  |
|  | IL-1β | F | GGGCCTCAAAGGAAAGAATCT |  |
|  |  | R | GAGGTGCTGATGTACCAGTTGG |  |
|  | IL-8 | F | CCTGCTGGCTGTCCTTAACC |  |
|  |  | R | TACAGAAGCTTCATTGCCGGT |  |
|  | hGAPDH | F | AAGGTCGGAGTCAACGGATTTG |  |
|  |  | R | CCATGGGTGGAATCATATTGGAA |  |
|  | HBV 3.5kb mRNA | F | GCCTTAGAGTCTCCTGAGCA |  |
|  |  | R | GAGGGAGTTCTTCTTCTAGG |  |
|  | HBV DNA | F_2150_ | CCTAGTAGTCAGTTATGTCAAC |  |
|  |  | R_2300_ | TCTATAAGCTGGAGGAGTGCGA |  |
|  | HBV cccDNA | F | CTCCCCGTCTGTGCCTTCT |  |
|  |  | R | GCCCCAAAGCCACCCAAG |  |
|  |  | Probe | FAM-ACGTCGCATGGAGACCACCGTGAACGCC-TAMRA |  |
| Primers for  off-target Assay | h off-1 | F | GCATGTGATCAGCCATGTAT |  |
|  |  | R | TGAGGTTCTAGGTAGCACAG |  |
|  | h off-2 | F | GTGTTTCCTCGAGGTCCAGG |  |
|  |  | R | GACGAGTTTTGGCTACCCCA |  |
|  | h off-3 | F | GCAAAATGCTGTGAGGCACA |  |
|  |  | R | GCCACCTGGTTCCTTCATGA |  |
|  | h off-4 | F | AGGTTCTTCCAGGCTCCTCT |  |
|  |  | R | CGGCCTCCATGTTAGTGGTT |  |
|  | h off-5 | F | GCCCACTCTTCCTTCTCCAC |  |
|  |  | R | ACAGCCCTGGGGTTTCAAAA |  |
|  | h off-6 | F | AACACAGGACCTGTCTGGGA |  |
|  |  | R | ACGTGTCTTTGCTCAGCAGA |  |
|  | h off-7 | F | TCCCCAACTAAATCATCAACCT |  |
|  |  | R | TCATCACAGCTGCCGTGATT |  |
|  | m off-1 | F | TGATCTTTCCTGTCTGCCCG |  |
|  |  | R | TTTCCCTTCATCCAGTGCCC |  |
|  | m off-2 | F | AGCTAGATCTGAAGGACGGGA |  |
|  |  | R | CCACAGGCAGAAGGACAGAG |  |
|  | m off-3 | F | TCTCCTGCATGATCCCCAGA |  |
|  |  | R | AAAACAACATGCCACCCTGC |  |
|  | m off-4 | F | AAGCCCAGGATCCAACTGTG |  |
|  |  | R | CCTTCCGCGATGACGGATAA |  |
|  | m off-5 | F | TCCATGCTCGGACATGGAAC |  |
|  |  | R | GAGGCCCCTTGAACAGACTC |  |
|  | m off-6 | F | GAGGTGCTGTAGCTGTCCAG |  |
|  |  | R | CACCCTGGGGAACAAGTTAGT |  |

h:human. m:mouse.
